# Microglial circ-UBE2K exacerbates depression by regulating parental gene UBE2K via targeting HNRNPU

**DOI:** 10.7150/thno.96890

**Published:** 2024-07-01

**Authors:** Yujie Cai, Yao Ji, Yingxuan Liu, Dandan Zhang, Zheng Gong, Li Li, Xiongjin Chen, Chunmei Liang, Sifan Feng, Jiongtong Lu, Qinjie Qiu, Zhixiong Lin, Yan Wang, Lili Cui

**Affiliations:** 1 Institute of Neurology, Guangdong Key Laboratory of Age-Related Cardiac and Cerebral Diseases, Affiliated Hospital of Guangdong Medical University, Zhanjiang, 524001, China.; 2 School of Ocean and Tropical Medicine, Guangdong Medical University, Zhanjiang, Guangdong, 524023, China.; 3 Institute of Laboratory Animal Center, Guangdong Medical University, Zhanjiang, China.; 4 Department of Psychiatry, Affiliated Hospital of Guangdong Medical University, Zhanjiang, China.

**Keywords:** circular RNA UBE2K (circ-UBE2K), major depressive disorder (MDD), microglial activation, neuroinflammation, heterogeneous nuclear ribonucleoprotein U (HNRNPU)

## Abstract

**Background:** Knowledge about the pathogenesis of depression and treatments for this disease are lacking. Epigenetics-related circRNAs are likely involved in the mechanism of depression and have great potential as treatment targets, but their mechanism of action is still unclear.

**Methods:** Circular RNA UBE2K (circ-UBE2K) was screened from peripheral blood of patients with major depressive disorder (MDD) and brain of depression model mice through high-throughput sequencing. Microinjection of circ-UBE2K overexpression lentivirus and adeno-associated virus for interfering with microglial circ-UBE2K into the mouse hippocampus was used to observe the role of circ-UBE2K in MDD. Sucrose preference, forced swim, tail suspension and open filed tests were performed to evaluate the depressive-like behaviors of mice. Immunofluorescence and Western blotting analysis of the effects of circ-UBE2K on microglial activation and immune inflammation. Pull-down-mass spectrometry assay, RNA immunoprecipitation (RIP) test and fluorescence *in situ* hybridization (FISH) were used to identify downstream targets of circ-UBE2K/ HNRNPU (heterogeneous nuclear ribonucleoprotein U) axis.

**Results:** In this study, through high-throughput sequencing and large-scale screening, we found that circ-UBE2K levels were significantly elevated both in the peripheral blood of patients with MDD and in the brains of depression model mice. Functionally, circ-UBE2K-overexpressing mice exhibited worsened depression-like symptoms, elevated brain inflammatory factor levels, and abnormal microglial activation. Knocking down circ-UBE2K mitigated these changes. Mechanistically, we found that circ-UBE2K binds to heterogeneous nuclear ribonucleoprotein U (HNRNPU) to form a complex that upregulates the expression of the parental gene ubiquitin conjugating enzyme E2 K (UBE2K), leading to abnormal microglial activation and neuroinflammation and promoting the occurrence and development of depression.

**Conclusions:** The findings of the present study revealed that the expression of circUBE2K, which combines with HNRNPU to form the circUBE2K/HNRNPU complex, is increased in microglia after external stress, thus regulating the expression of the parental gene UBE2K and mediating the abnormal activation of microglia to induce neuroinflammation, promoting the development of MDD. These results indicate that circ-UBE2K plays a newly discovered role in the pathogenesis of depression.

## Introduction

Major depressive disorder (MDD), a chronic disease that is caused mainly by genetic and environmental factors, is characterized by emotional disorder and seriously threatens human health 1, 2. It is expected that by 2030, more than 350 million people worldwide will suffer from depression, which is associated with high disability and suicide rates 3. Epidemiological studies show that long-term exposure to stressful life events is considered the main risk factor for depression. At present, studies on the pathogenesis of depression, including genetic, biochemical, immunological, neuroendocrinological, electrophysiological, neurostructural, psychosocial and epigenetic factors, have attracted much attention 4, 5. However, due to the heterogeneity of MDD cases, the etiology and pathogenesis of MDD have not been thoroughly elucidated. Therefore, further study of the detailed mechanism of MDD could help to identify more effective therapeutic targets.

CircRNAs are noncoding RNAs with closed loop structures that are widely expressed in mammalian tissues 6, 7. CircRNAs with closed loop structures are more stable and have longer half-lives than other noncoding RNA molecules (such as miRNAs and lncRNAs) and can resist degradation by ribonuclease R (RNase R); thus, they are ideal diagnostic biomarkers. In recent years, important studies have shown that circRNAs play key roles in intercellular communication, neural cell morphogenesis, plasticity and nervous system-related diseases 8-11. CircRNAs exhibit dynamic expression patterns under a series of physiological and pathological conditions and play important roles in regulating gene transcription, protein expression, DNA methylation and peptide translation 12. Our previous transcriptome analysis and other studies indicated that circRNAs are abnormally expressed in the peripheral blood of MDD patients and depression model animals 12-15, suggesting that circRNAs could be novel biomarkers of MDD pathogenesis and/or new therapeutic targets. Although thousands of abnormally expressed circRNAs have been found, only a few of their functions have been elucidated.

The neuroinflammatory response is one of the core pathological mechanisms in many central nervous system diseases 16, 17. Inflammation is closely related to the onset of depression. Inflammation in the central nervous system is one of the important pathophysiological mechanisms leading to the occurrence of depression 18. In recent years, increasing evidence has shown that microglia sense depression-related stressors and trigger immune responses and neuroinflammation, leading to dysfunction. This dysfunction may play a role in disrupting neurological function, leading to depression 19, 20. In this study, we focused on circ-UBE2K, which is abnormally expressed in the peripheral blood of MDD patients and is conserved between humans and mice. By studying changes in the peripheral blood of MDD patients and depression model animals together with changes in the expression of circ-UBE2K *in vivo* and *in vitro*. The FISH-IF results showed that circ-UBE2K had the greatest expression in microglia in the mouse brain. Increasing the expression of circ-UBE2K aggravated neuroinflammation and neuronal damage. Then, we confirmed the function of circ-UBE2K and the specific mechanism by which it binds to the HNRNPU protein to regulate UBE2K protein expression and mediate abnormal microglial activation to promote the development of depression. Our findings link the epigenetic regulation of depression with abnormal activation of microglia and circ-UBE2K, providing evidence that circUBE2K may be a new therapeutic target for MDD.

## Results

### Upregulation of circ-UBE2K in MDD patients and depression model animals

**Figure 1:**
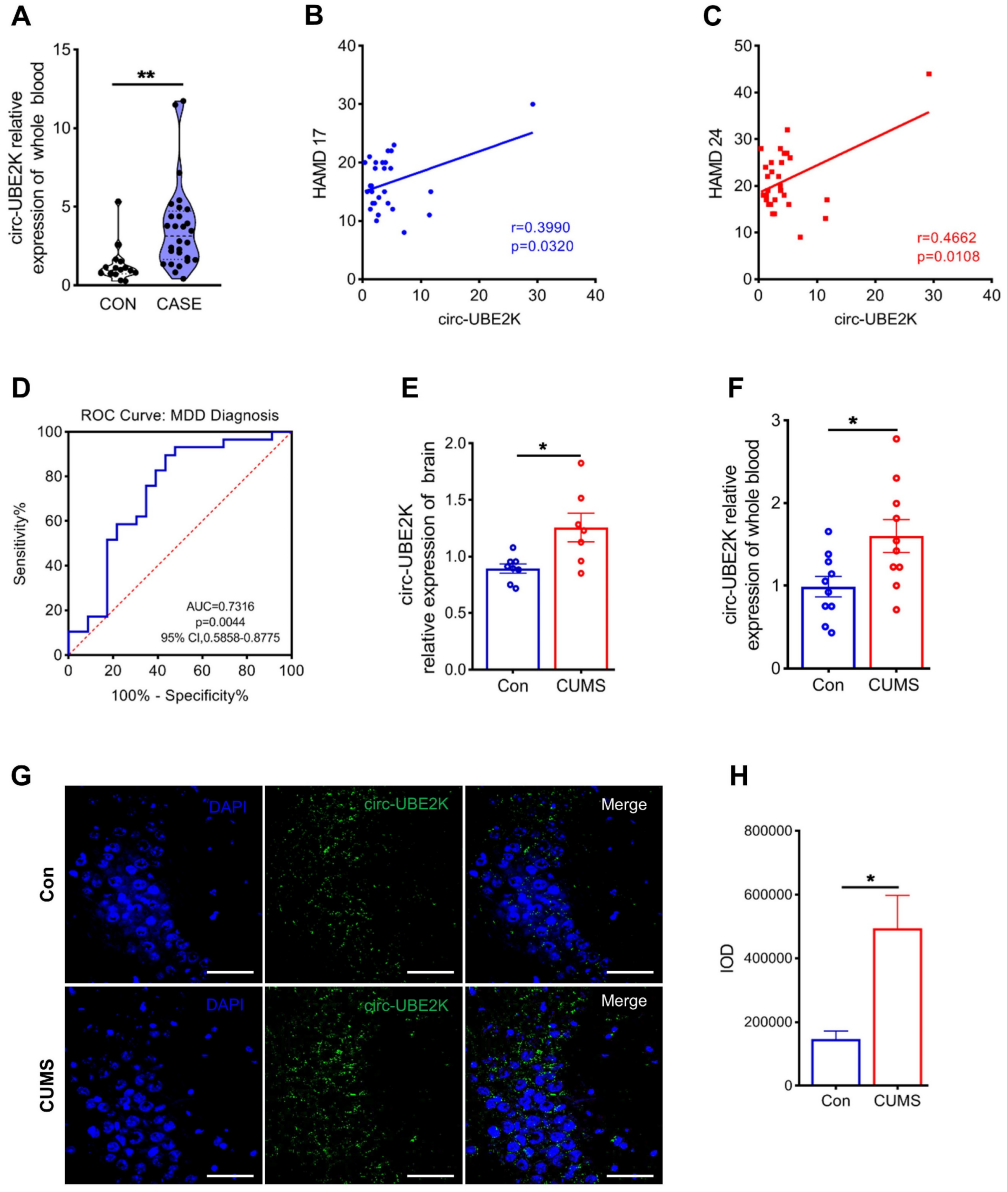
** Circ-UBE2K was significantly upregulated in MDD patients and depression model mice.** (A) qPCR analysis of circ-UBE2K expression levels in the peripheral blood of MDD patients (n=29) and healthy control subjects (n=16). (B) Correlations between circ-UBE2K expression and HAMD-17 scores according to Pearson's correlation coefficient. (C) Correlations between circ-UBE2K expression and HAMD-24 scores were determined using Pearson's correlation coefficient. (D) ROC curve for circ-UBE2K expression levels in patients with MDD and healthy control subjects. (E) qPCR analysis of circ-UBE2K expression levels in the brain tissues of CUMS model mice (n =7) and control mice (n =8). (F) qPCR analysis of circ-UBE2K expression levels in the whole blood of CUMS model mice (n =10) and control mice (n =10). (G-H) Images of circ-UBE2K (G) and quantification of circ-UBE2K levels (H) in brain tissues from CUMS model mice and control mice. Green, FITC-labeled probes specific for circ-UBE2K; blue, DAPI (nuclei); the merged image shows overlap of the fluorescent signals. Scale bar, 50 µm. The data are presented as the mean ± SEM. *P < 0.05 and **P < 0.01.

Previously, using whole-transcriptome sequencing, we analyzed blood samples from four MDD patients and four healthy controls and identified 445 differentially expressed circRNAs 13. Afterward, we used the circRNADB, CIRCpedia, circBase and circAltas databases to screen circRNAs with high homology between humans and mice and identified 12 circRNAs with high homology (sequence similarity ≥ 85%) between these species (**Figure S1A**). We used qPCR to evaluate the expression levels of these homologous circRNAs in the peripheral blood of MDD patients and normal controls. The results showed that circ-UBE2K and circ-TTC8 were highly expressed in the peripheral blood of MDD patients (**Figure 1A and Figure S1B-D**). Then, we examined the correlation between the circ-UBE2K and circ-TTC8 expression level in peripheral blood and scores on the Hamilton Depression Scale (HAMD-17 and HAMD-24) in patients with depression. The results showed that the circ-UBE2K level was positively correlated with the HAMD-17 score (Pearson correlation coefficient, r=0.3990; *p*=0.0320) and HAMD-24 score (Pearson correlation coefficient, r=0.4662; *p*=0.0108), however, the level of circ-TTC8 is not correlated with the HAMD-17 and HAMD-24 scores. (**Figure 1B-C and Figure S1E-F**). Moreover, the area under the receiver operating characteristic (ROC) curve of circ-UBE2K expression was 0.7316 (*P*=0.0044, **Figure 1D**). Therefore, we selected circ-UBE2K and further analyzed its potential role in MDD.

Circ-UBE2K (hsa_circ_0009154) is located on chromosome 4 and consists of exons 2, 3 and 4. Sequence alignment analysis using a clone manager showed that human, mouse circ-UBE2K have 97% homology and that the ring splicing sites are identical (**Figures S2A-B**). This indicated that circ-UBE2K is a highly conserved circular RNA between humans and mice. Therefore, we constructed a chronic unpredictable mild stress (CUMS)-induced depression mouse model and measured the levels of circ-UBE2K in the brain tissue and peripheral blood of depression model mice and normal control mice (**Figures S2C-I**).

We evaluated the growth of mice. The weight of the mice was not significantly influenced during exposing to the CUMS protocol for 4 weeks (**Figure S2D**). Open filed test (OFT) has performed in depression model mice and normal control mice. The results of the open field test did not reveal a noticeable difference in total distance and number of standing among the different groups (**Figure S2E-F**). These indicated that depression-like mice did not exhibit anxiety-like behaviors. Consistent with the data from the clinical peripheral blood samples, circ-UBE2K expression levels increased in the peripheral blood and brain tissues of CUMS model mice compared with those of control mice (**Figure 1E-F**). In addition, RNA-fluorescence *in situ* hybridization (RNA-FISH) showed that circ-UBE2K was highly expressed in the CA3 region of the mouse hippocampus (**Figure 1G-H and Figure S3**). We also verified that there was no significant difference in the expression of circ-UBE2K in organs other than the brain and blood, such as the heart, liver, spleen and kidney (**Figure S2J-M**). While circ-UBE2K was significantly reduced in the lung (**Figure S2N**).

### Characterization and distribution of circ-UBE2K in mouse brain tissues

**Figure 2:**
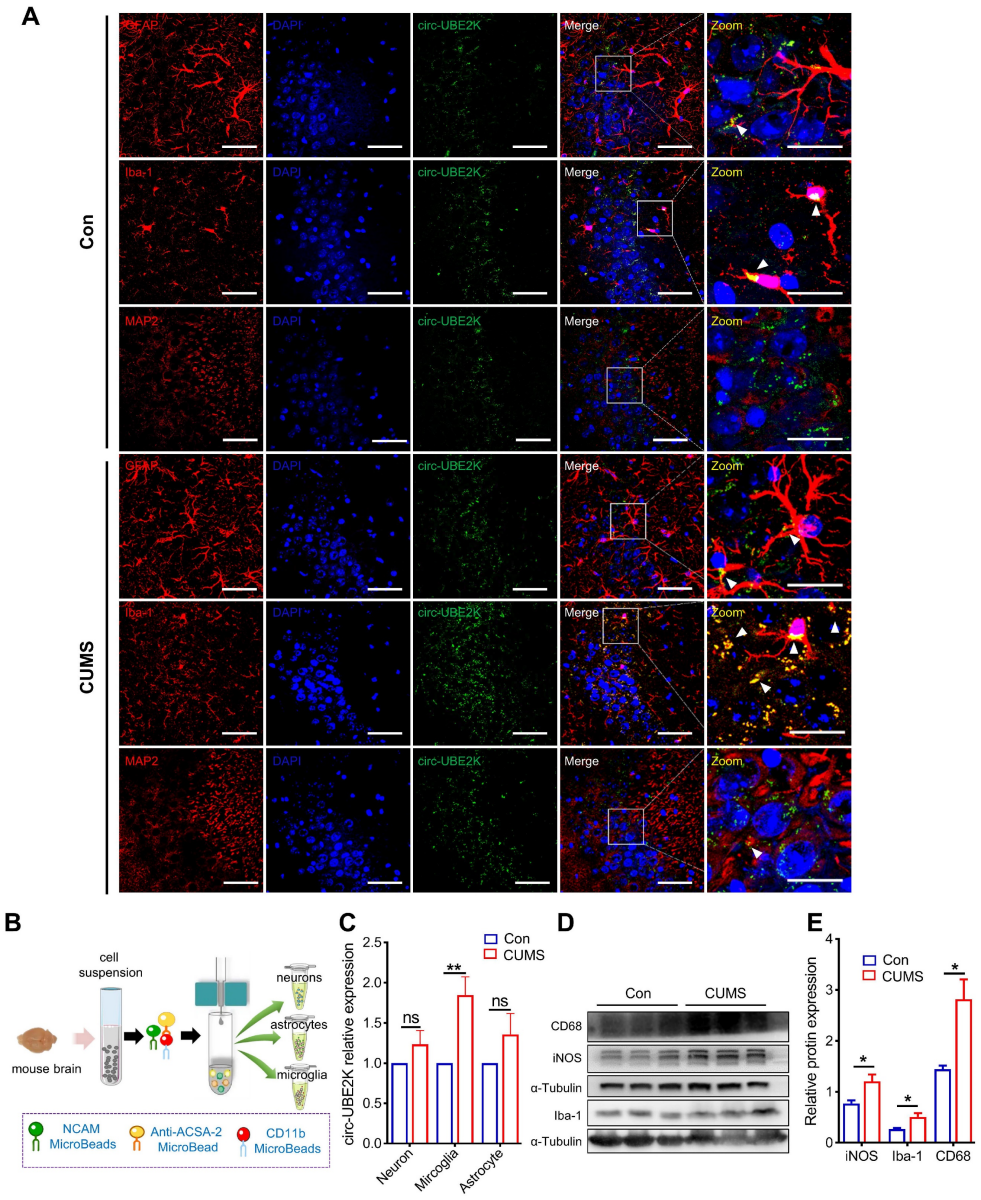
** Expression and distribution of circ-UBE2K in mouse brain tissues.** (A) Distribution of circ-UBE2K in different cell types in the mouse brain CA3 region. The white triangular arrow points to the co-localization of circ-UBE2K with astrocytes, microglia and neurons. Scale bar, 50 µm (overview) and 20 µm (overview). (B) Schematic diagram of the magnetic bead method used to isolate neurons, astrocytes, and microglia in the mouse brain. (C) qPCR analysis of circ-UBE2K expression in neurons, astrocytes, and microglia from CUMS model mice (n=4) and control mice (n=4), which were separated by magnetic beads. (D-E) Western blot analysis of Iba-1, iNOS and CD68 expression levels in CUMS model mice (n=3) and control mice (n=3). All the data are presented as the mean ± SEM. One-way ANOVA followed by Tukey's post hoc test. ns, not significant, *P < 0.05 and **P < 0.01.

**Figure 3:**
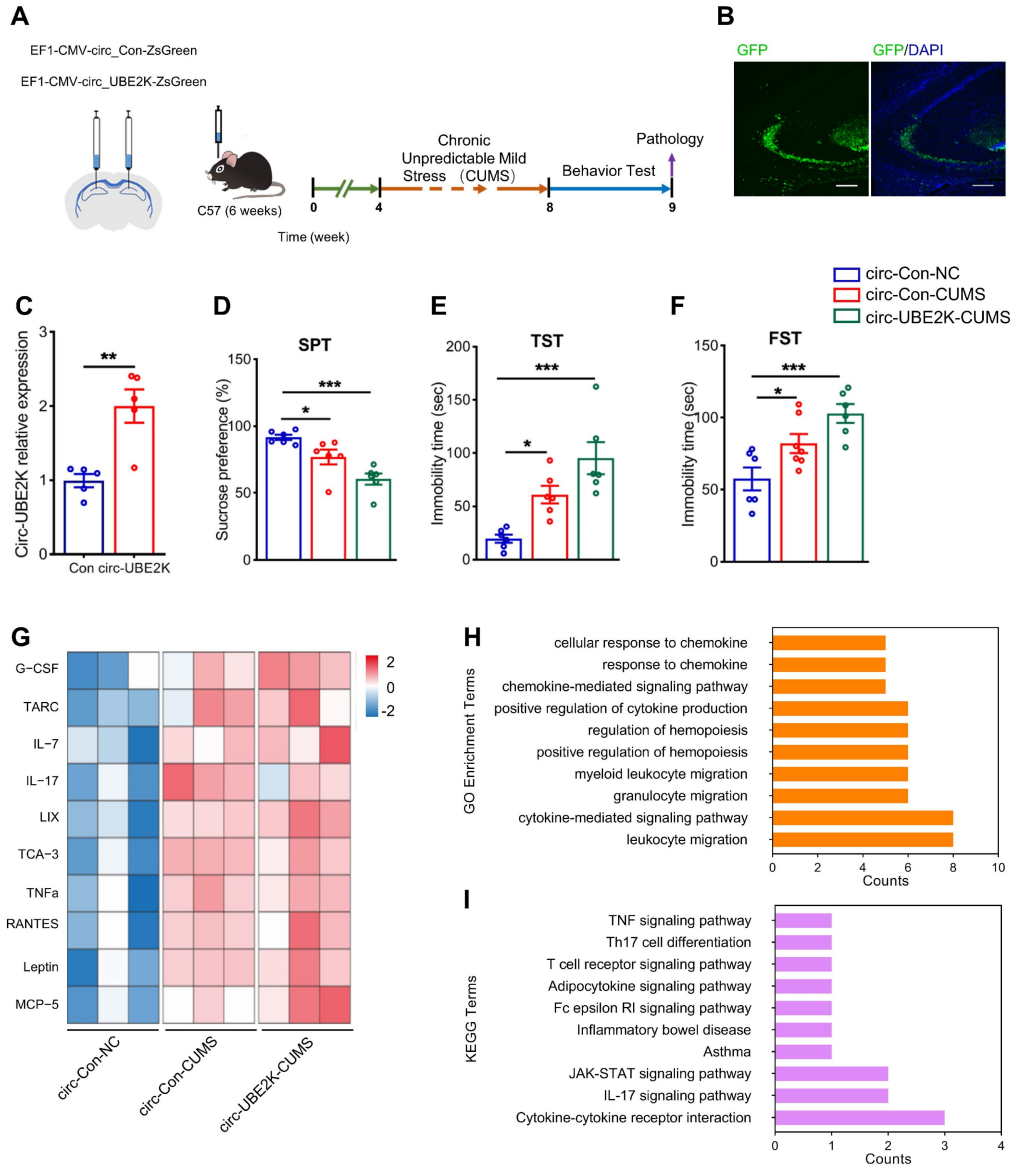
** Overexpression of circ-UBE2K aggravated depression-like behavior and inflammation.** (A) Illustration of bilateral stereotactic injection of lentivirus into CUM-induced depression model mice and timeline of the experimental procedure. Six-week-old mice were injected with lentivirus into the hippocampus. (B) The success of the injection was assessed by assessing GFP expression 4 weeks after microinjection. (C) Circ-UBE2K levels were measured 4 weeks after microinjection. n =5/group. (D-F) Effects of microinjection of a circ-UBE2K-overexpressing lentivirus on depression-like behavior in CUMS model mice. Performance in the SPT (D), TST (E) and FST (F) was evaluated 4 weeks after CUMS exposure. n =6/group. (G) Heatmaps of differentially expressed immune and inflammatory cytokines identified by a semiquantitative cytokine array (Quantibody® Mouse Inflammation Array 1). n = 3 mice/group. The color indicates the degree of upregulation (red tones) or downregulation (magenta tones) in the circ-Con-NC group, circ-Con-CUMS group and circ-UBE2K-CUMS group. (H-I) GO enrichment analysis and KEGG enrichment analysis of the DEPs. All the data are presented as the mean ± SEM. *P < 0.05, **P < 0.01 and ***P < 0.001. Unpaired Student's t-test for C, One-way ANOVA followed by Tukey's post hoc test for D-F.

To further assess which cells in the brain predominantly express circ-UBE2K, *in situ* hybridization was performed, and the results showed that more circ-UBE2K was derived from microglia than from astrocytes and neurons (**Figure 2A**). We further evaluated whether circ-UBE2K in these cells was upregulated in the brains of depression model mice. We compared the circ-UBE2K levels in the lysates of neurons (NCAM-positive), astrocytes (ACSA-2-positive) and microglia (CD11b-positive) isolated from the brain tissues of CUMS model and control mice (**Figure 2B**). qPCR revealed that circ-UBE2K was significantly upregulated in microglia compared with neurons and astrocytes (**Figure 2C**). Since the activation of microglia and neuroinflammation may mediate the onset of depression, we then examined the changes in the expression of CD68 and iNOS and the expression of inflammatory cytokines 20, 21. As shown in **Figure 3D-E**, the expression levels of iNOS, CD68 were increased in CUMS model mice.

### Circ-UBE2K overexpression aggravates depressive-like Behaviors induced by CUMS

Next, we studied the effect of circ-UBE2K on CUMS model mice. We microinjected a circ-Control ZsGreen or circ-UBE2K-ZsGreen lentivirus into the bilateral hippocampus (**Figure 3A**). Four weeks after injection, the green fluorescent protein (GFP)-carrying lentiviruses were expressed in the hippocampus (**Figure 3B and Figure S4**). Compared with that in the mice injected with circ-Control, the expression of circ-UBE2K in the mice injected with circ-UBE2K increased (**Figure 3C**). Four weeks after microinjection of the lentiviruses, the mice were exposed to the CUMS protocol for 4 weeks. We tested the growth status of mice and found that circ-UBE2K had no significant effect on their growth (**Figure S5A**). Then, we assessed depressive-like behavior 4 weeks later. The results of the sucrose preference test (SPT) showed that compared with the control group, CUMS model mice exhibited a reduced sucrose preference, overexpression of circ-UBE2K aggravated this reduction (**Figure 3D**). The forced swimming test (FST) and tail suspension test (TST) were also conducted to evaluate the effect of circ-UBE2K on depressive-like behavior. As shown in **Figure 3E** (TST) and **Figure 3F** (FST), the immobility time of CUMS model mice was significantly greater than that of control mice, and this increase was significantly aggravated after microinjection of circ-UBE2K. In addition, the OFT results showed that there were no significant differences in the total distance and number of standing times of the groups of mice (**Figure S5B-C**). Subsequently, changes in the levels of 40 cellular immune and inflammatory factors in the hippocampus of mice subjected to CUMS after microinjection of circ-UBE2K were assessed with a Quantibody Mouse Inflammation Array 1. Heatmaps of the differentially expressed proteins (DEPs) are shown in **Figure 3G**. The results of Gene Ontology (GO) enrichment analysis and KEGG enrichment analysis of the DEPs are shown in **Figure 3H-I**. According to the GO enrichment analysis results, the main biological process terms in which the DEPs were enriched were cytokine activity (GO:0005125), receptor ligand activity (GO:0048018), cytokine receptor binding (GO:0005126) and chemokine activity (GO:0008009) (**Figure 3H**). In addition, KEGG enrichment analysis (**Figure 3I**) revealed that the DEPs were enriched in the TNF signaling pathway (mmu04668), the IL-17 signaling pathway (mmu04657) and cytokine‒cytokine receptor interaction (mmu04060).

### Circ-UBE2K overexpression promotes microglial activation and neuroinflammation

The excessive activation of microglia leads to neuroinflammation, neuronal activity and concomitant behavioral changes, including anxiety and depression 19. In addition, circ-UBE2K expression is increased in the microglia of CUMS model mice. Thus, we next examined the effects of circ-UBE2K on microglial activation and hippocampal morphology. First, we analyzed the morphology of the microglia because of it is closely related to their activation status 22. Microglial branches were skeletonized and quantified using ImageJ.

**Figure 4:**
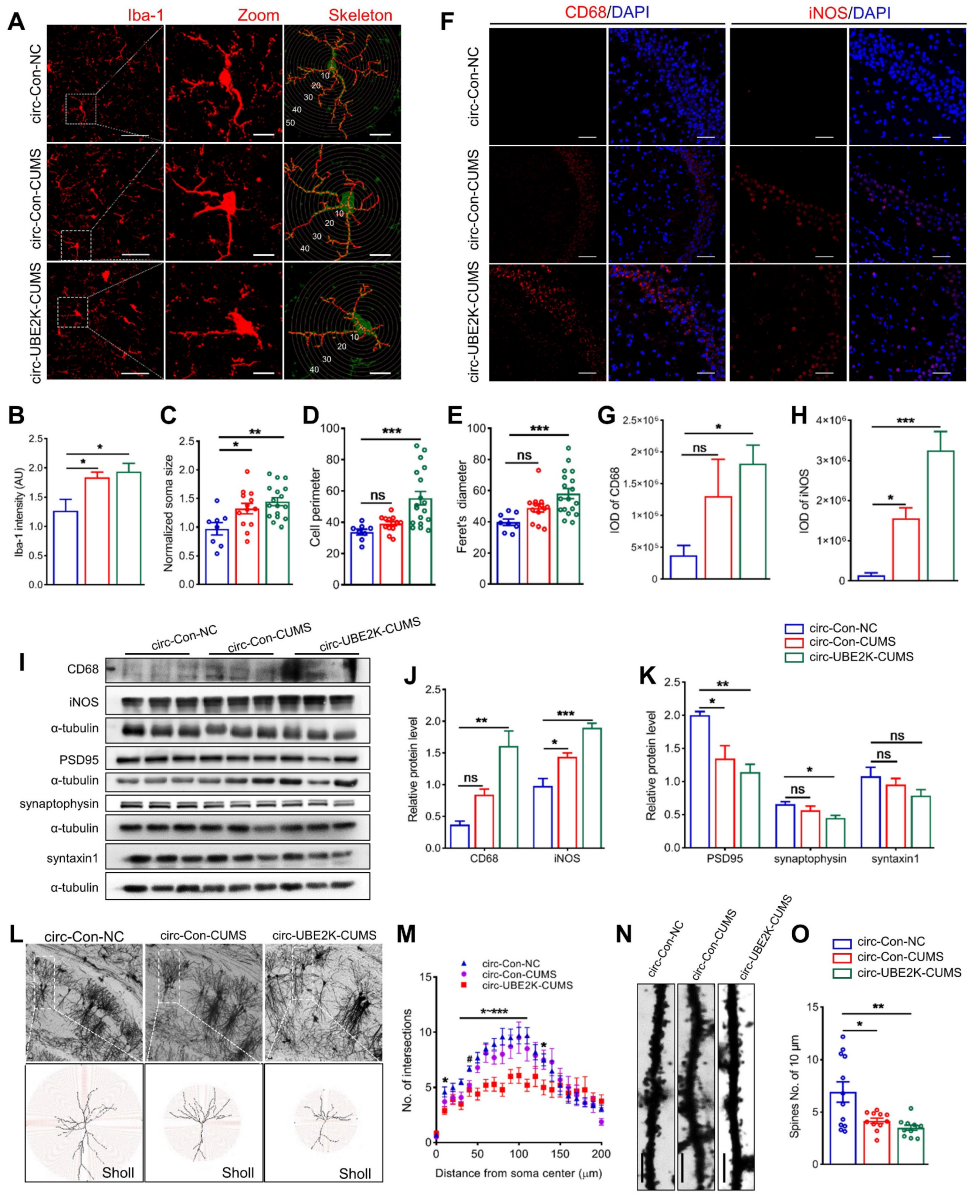
** Circ-UBE2K overexpression promote glial activation and neuronal damage.** (A) Immunofluorescence staining of Iba1-positive cells in the hippocampus in the different groups. Scale bars, 50 μm. Magnified images are shown in the middle column, and skeletal diagrams of Iba1-positive cells are shown on the right of panel A. Scale bars, 10 μm. (B-E) Quantification of the Iba1intensity, soma volume, branch length and feret's diameter of cells. (F-H) Representative images of iNOS and CD68 immunostaining (F) and quantification of iNOS and CD68 levels (G-H) in the hippocampus in the different groups. Scale bars, 50 μm. (I-K) Representative Western blots of the CD68, iNOS, PSD95, synaptophysin and syntaxin1 expression (I) and quantification of these proteins expression levels (J-K) in the different groups. (L) Representative microphotographs of the Golgi-stained hippocampus (up) and skeletonized CA1 pyramidal neurons in the hippocampus (down) in the different groups. Scale bars, 50 μm. (M) Sholl analysis of the dendritic complexity of CA1 pyramidal neurons in the different groups. 10 neurons /group. ^#^*P* < 0.05 circ-Con-NC vs circ-Con-CUMS, *P < 0.05, **P < 0.01 and ***P <0.001 circ-Con-NC vs circ-UBE2K-CUMS. (N-O) Representative images of neuronal dendrites after Golgi-Cox staining in the different groups (N) and summary data for the spine number/10 μm (O). All the data are presented as the mean ± SEM. ns, not significant, *P < 0.05, **P < 0.01 and ***P < 0.001.

Compared with that of microglia in the control group, the length of branches decreased significantly, circ-UBE2K overexpression enhanced these effects (**Figure 4A**), the Iba-1 intensity, somatic cell size, cell perimeter and feret's diameter of microglia in the CUMS-exposed mice increased, and circ-UBE2K overexpression enhanced these effects (**Figure 4B-E**). In addition, we evaluated microglial activation and neuroinflammation in CUMS-exposed mice after overexpression of circ-UBE2K by measuring the expression of iNOS and CD68. As shown in** Figure 4F-I**, CUMS increased the expression of iNOS and CD68 in mice, and overexpression of circ-UBE2K significantly aggravated the increase in the expression of these proteins induced by CUMS (**Figure 4J**).

Microglia are resident macrophages of the central nervous system, with various forms and functions. Functional deficits caused by abnormal activation of microglia may lead to synaptic abnormalities in aging, Alzheimer's disease, traumatic brain injury, HIV related neurocognitive disorders, and other neurological or psychiatric disorders such as autism, depression, and post-traumatic stress disorder 22-24. To explore the potential link between circ-UBE2K mediated abnormal activation of microglia leading to neuroinflammation and neuronal synaptic disorders, we used Golgi staining to assess dendritic complexity and spine density of pyramidal cells in the CA1 region (**Figure 4L**). Dendritic branches were skeletonized and dendritic arborizations were quantified using Fiji (**Figure 4L**). The results showed that pyramidal cells from circ-UBE2K CUMS model mice exhibited a less complex branching pattern than those from control group mice in regions 40 -110μm from the centre of soma (**Figure 4M**). The number of dendritic spines on the neurons of CUMS model mice decreased, and the overexpression of circ-UBE2K aggravated this decrease (**Figure 4N-O**). Next, we quantified the levels of synaptic proteins in the hippocampus. Western blot analysis of synaptic proteins revealed that the protein expression levels of synaptophysin (presynaptic protein), PSD95 (postsynaptic protein) and syntaxin1 (synaptic fusion protein) were reduced in CUMS model mice, circ-UBE2K overexpression aggravated these changes (**Figure 4I and K**).

### Microglia-specific circ-UBE2K inhibition attenuates depressive-like behavior and reverses microglial activation in mice

**Figure 5:**
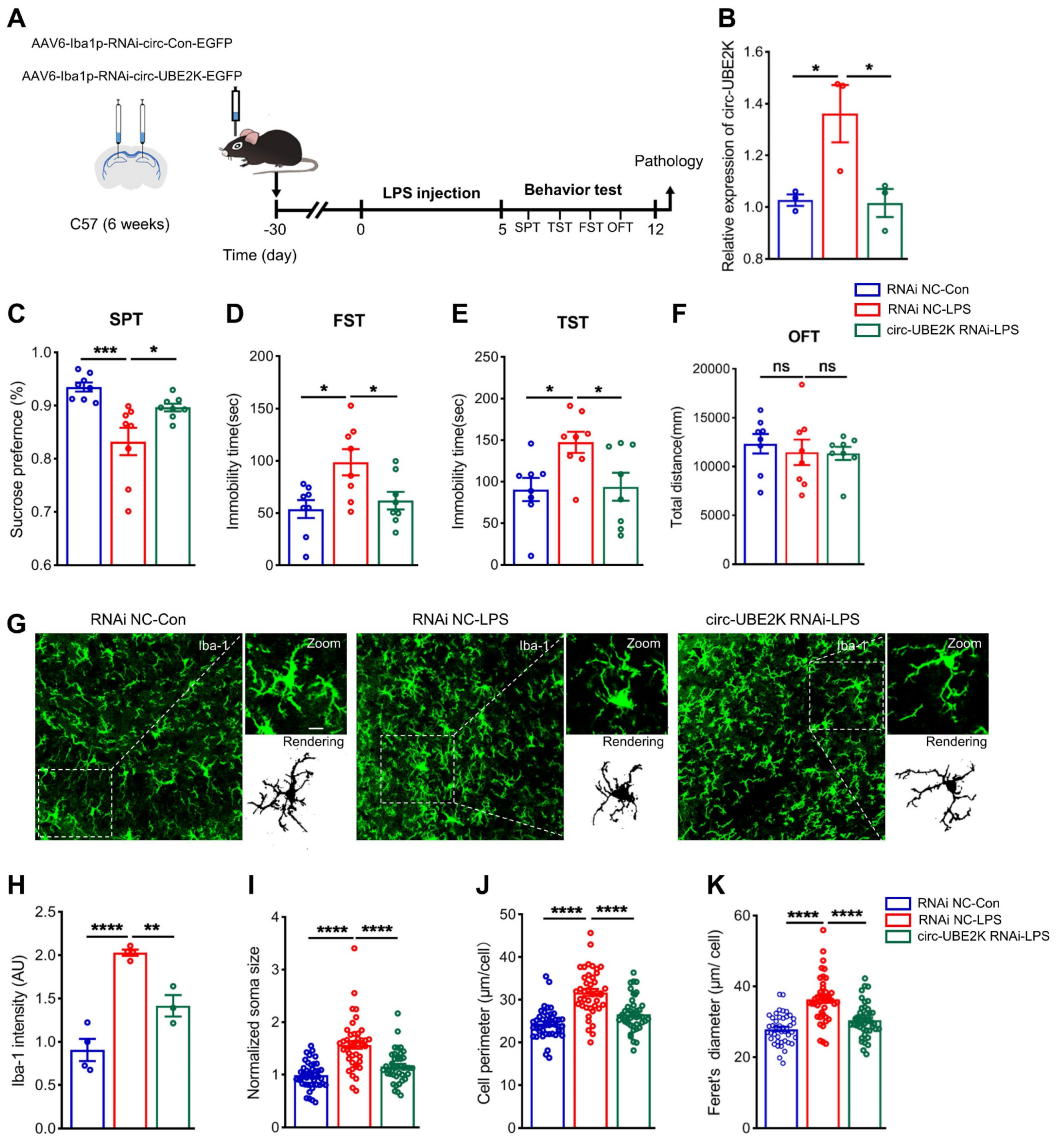
** Specific interference with circ-UBE2K expression in microglia mitigates depressive behavior in mice and normalizes microglial activation.** (A) Illustration of bilateral stereotactic injection of AAV into LPS-induced depression model mice and timeline of the experiment. Six-week-old mice were injected with AAV into the hippocampus. (B) Circ-UBE2K levels were measured 4 weeks after microinjection and 5 days after LPS treatment. n =3/group. (C-F) Results of the behavioral tests (SPT, FST, TST, and OFT) for the different groups. (G) Representative images of Iba1 (green) immunostaining and 3D reconstruction (black) of microglia in the different groups. Scale bars, 50 μm (overview) and 10 μm (inset and rendering). (H-K) Quantification of the Iba-1 intensity, soma volume, branch length and feret diameter of Iba1-positive cells. All the data are presented as the mean ± SEM. ns, not significant, *P < 0.05, **P < 0.01, ***P < 0.001 and ****P < 0.0001. One-way ANOVA followed by Tukey's post hoc test.

To further confirm the role of circ-UBE2K in depression model mice, we specifically knocked down circ-UBE2K in microglia to determine whether inhibition of circ-UBE2K can attenuate depression-like behavior in mice. LPS is widely considered to induce depression-like behavior in mice and is used to activate microglia 25, 26. 4 weeks after an adeno-associated virus for interfering with microglial circ-UBE2K (AAV6-Iba1-RNAi-circ-UBE2K) was injected into the bilateral hippocampus of the mice via a stereotaxic technique, we intraperitoneally injected the mice with LPS (1 mg/kg) or saline for five consecutive days (**Figure 5A**). First, we evaluated the expression of circ-UBE2K after LPS treatment. qPCR revealed that the circ-UBE2K level in the brain was significantly greater in mice in the LPS model group than in mice in the control group but decreased after microinjection of the circ-UBE2K RNAi virus (**Figure 5B**). Then, the mice were tested for depressive-like behavior. Compared with those in the control group, the mice in the LPS model group displayed depressive-like behaviors, including decreased sucrose preference and increased immobility time in the FST and TST. A reduction in circ-UBE2K expression ameliorated these changes (**Figure 5C-E**). The performance of the mice in the open field test (OFT) did not change (**Figure 5F**). Then, we evaluated the microglia status. Immunofluorescence analysis showed that microglial activation was observed in LPS-treated mice. Inhibition of circ-UBE2K attenuated microglia activation (**Figures 5G-K**).

### Decreased circ-UBE2K levels change the microglial phenotype in an LPS-treated cell model

To elucidate the effect of microglial circ-UBE2K on metabolic pathways, we performed RNA sequencing (RNA-seq) of brain samples from AAV6-Iba1-RNAi-circ-UBE2K and AAV6-Iba1-RNAi-Con group mice. Heatmaps of the differentially expressed genes (DEGs) are shown in **Figure S6A**. We performed GO enrichment analysis of biological process terms for the important DEGs between these groups. According to GO enrichment analysis, the main molecule functions in which the DEGs were enriched were “transmembrane receptor protein serine/threonine kinase binding”, “neuropeptide hormone activity”, “RNA polymerase II-specific DNA-binding transcription factor binding” and “DNA-binding transcription factor binding” (**Figure S6B**).

After confirming that circ-UBE2K increased microglial activation *in vivo*, we further transfected microglia with circ-UBE2K siRNA *in vitro* to evaluate the effect of circ-UBE2K on LPS-induced microglial activation (**Figure S6C**). We found that the levels of the proinflammatory cytokines IL-6, IL-1 and TNF-α were significantly increased in LPS-treated HMC3 microglia (**Figure S6D-F**). In addition, interfering with the expression of circ-UBE2K significantly inhibited the increase in the expression of proinflammatory factors induced by LPS (**Figure S6D-F**).

### Effect of circ-UBE2K on regulating the expression of its host gene UBE2K

**Figure 6:**
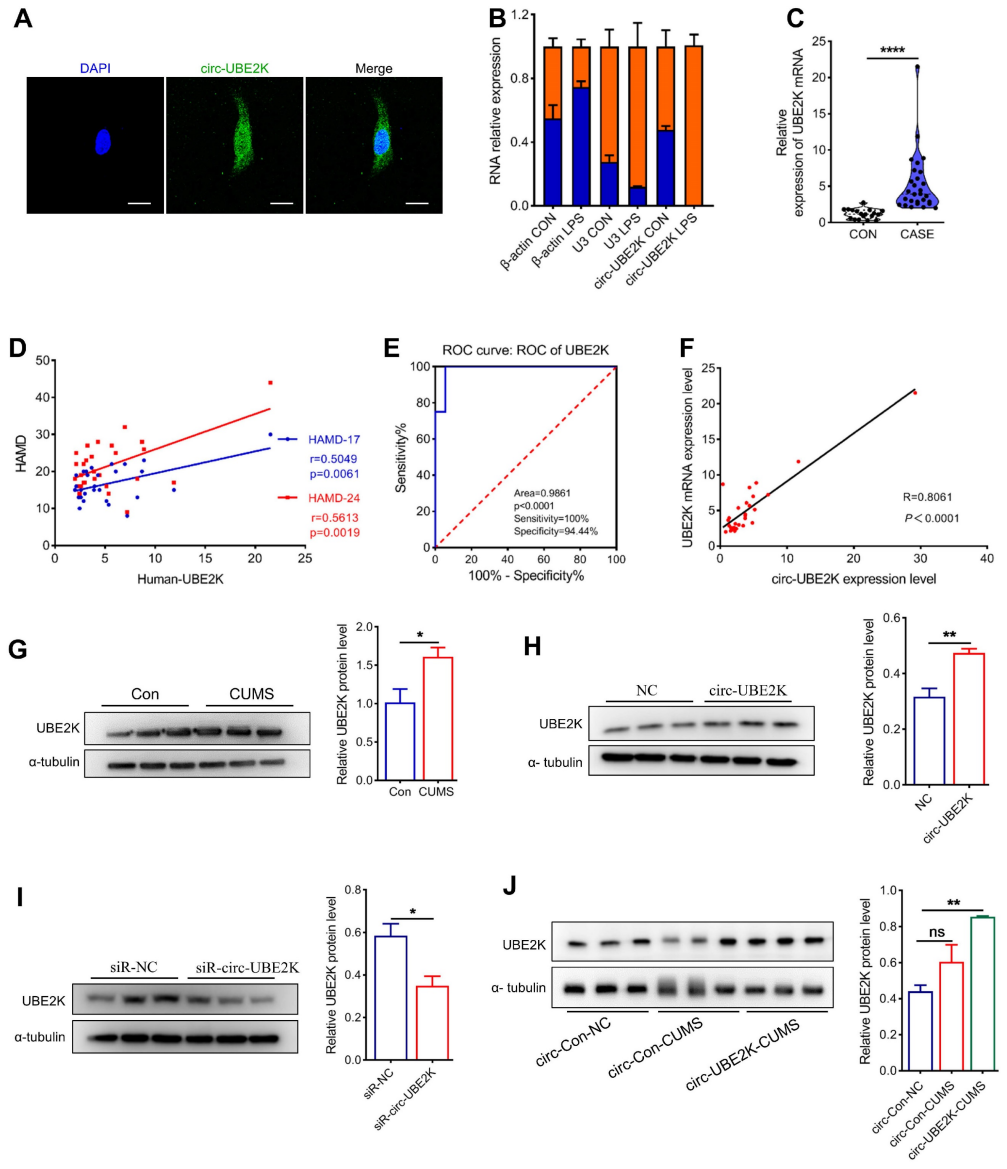
** Circ-UBE2K positively regulates its host gene UBE2K expression.** (A) The distribution of circ-UBE2K in HMC3 cells determined by FISH. FITC-labeled probes specific for circ-UBE2K. Scale bar, 20 μm. (B) The relative expression of circ-UBE2K was measured by qPCR after isolation of RNA from the cell nucleus and cytoplasm. (C) qPCR analysis of UBE2K mRNA expression levels in the peripheral blood of MDD patients (n=29) and healthy control subjects (n=16). (D) Correlations between UBE2K mRNA expression and HAMD-17 and HAMD-24 scores were determined using Pearson's correlation coefficient. (E) ROC curve for UBE2K mRNA expression levels in patients with MDD and healthy control subjects. (F) Correlations between circ-UBE2K expression and UBE2K mRNA expression were determined using Pearson's correlation coefficient. (G) Western blot analysis of UBE2K expression levels in CUMS model mice (n =3) and control mice (n =3). (H-I) Western blot analysis of UBE2K expression after transfection of cells with the circ-UBE2K overexpression plasmid or siRNAs. (J) Western blot analysis of UBE2K expression in the circ-Con-NC group, circ-Con-CUMS group and circ-UBE2K-CUMS group. All the data are presented as the mean ± SEM. ns, not significant, *P < 0.05, **P < 0.01 and ****P < 0.0001. Unpaired Student's t-test for C, G, H, I. One-way ANOVA followed by Tukey's post hoc test for J.

We further explored the molecular mechanism responsible for the effect of circ-UBE2K by determining whether it was localized in microglia via fluorescence *in situ* hybridization (FISH). The results showed that circ-UBE2K was expressed in both the cytoplasm and nucleus (**Figure 6A**). qPCR analysis of nuclear and cytoplasmic circ-UBE2K expression showed the same results (**Figure 6B**). After microglia were treated with LPS, circ-UBE2K was isolated from the cell nucleus and cytoplasm for qPCR analysis, and strikingly, the level of circ-UBE2K in the nucleus was significantly increased (**Figure 6B**). Based on reports that circRNAs may play different roles according to their subcellular localization, we speculated that circ-UBE2K may bind to proteins to participate in gene splicing or transcriptional regulation. The qPCR results showed that UBE2K mRNA was highly expressed in the peripheral blood of MDD patients and was positively correlated with HAMD-17 (Pearson correlation coefficient r=0.5049, p=0.0061) and HAMD-24 (Pearson correlation coefficient r=0.5613, p=0.0019) scores (**Figure 6C-D**). In addition, the area under the receiver operating characteristic (ROC) curve for UBE2K mRNA expression was 0.9861 (P < 0.0001, **Figure 6E**). A significant positive correlation was also found between the expression of circ-UBE2K and that of UBE2K in the peripheral blood of MDD patients (**Figure 6F**). Moreover, the protein expression of UBE2K was increased in the CUMS model group, which was consistent with the change in the UBE2K mRNA level in the clinical peripheral blood samples (**Figure 6G**).

Next, we investigated the effect of circ-UBE2K on the expression of its parent gene, UBE2K. Circ-UBE2K overexpression increased the UBE2K protein level (**Figure 6H**). Conversely, knockdown of circ-UBE2K decreased the protein level of UBE2K (**Figure 6I**). In addition, the level of the UBE2K protein in the brains of mice after microinjection of the circ-UBE2K-overexpressing lentivirus also increased (**Figure 6J**). Thus, we speculate that circ-UBE2K might exert its physiological functions by regulating the expression of its host gene UBE2K.

### Circ-UBE2K directly interacts with the HNRNPU protein to enhance UBE2K expression

**Figure 7:**
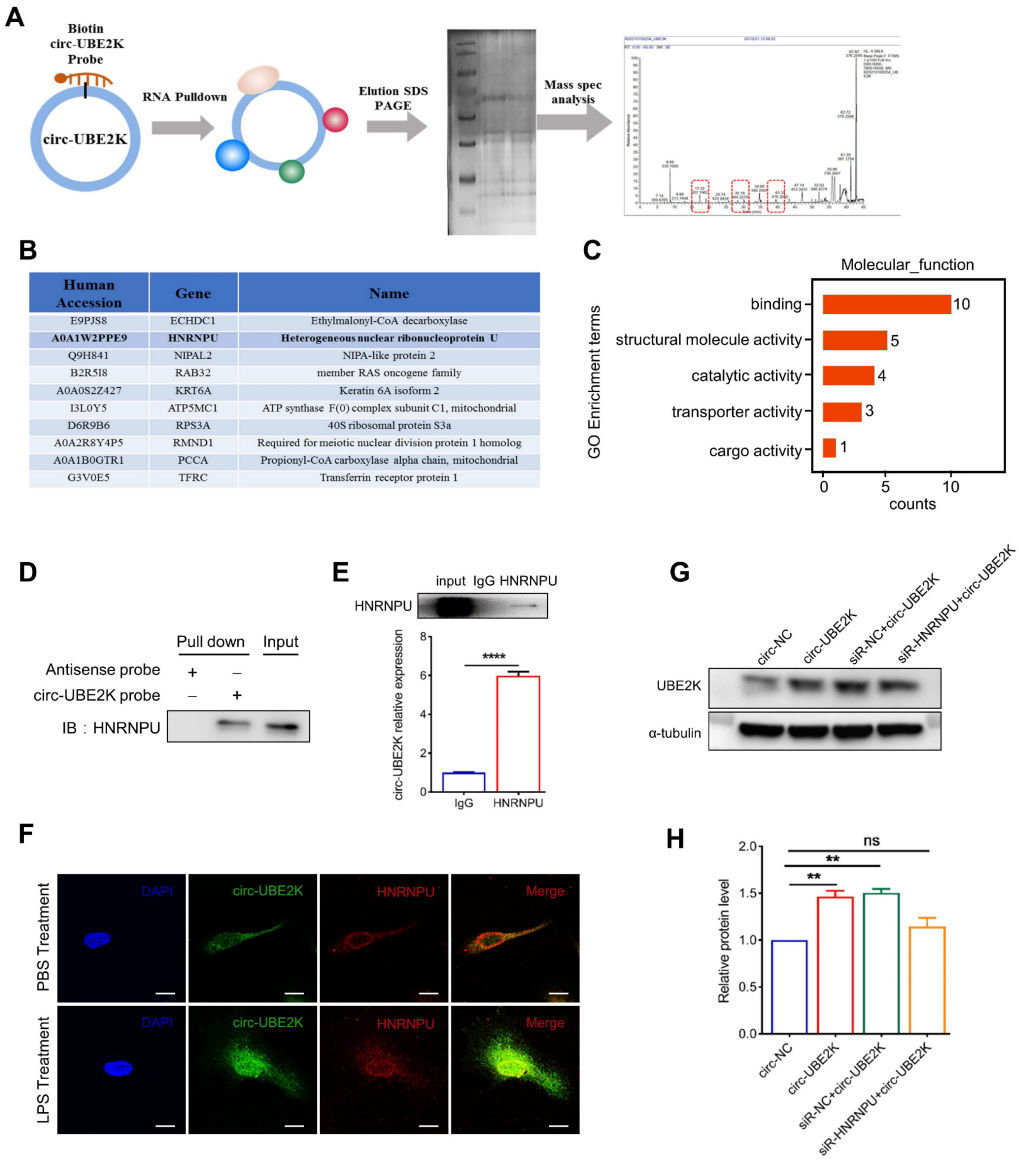
** Circ-UBE2K directly interacts with the HNRNPU protein in microglia.** (A) A biotin-labeled probe and a control (Ctrl) probe complementary to the circ-UBE2K junction were incubated with HMC3 cells. Circ-UBE2K-interacting proteins were identified by mass spectrometry after pull-down with streptavidin beads. (B) The top 10 proteins identified from the pull-down/mass spectrometry data. (C) GO enrichment analysis of the pulled down proteins. (D) Immunoblot analysis of HNRNPU after the pulldown assay showing its specific association with circ-UBE2K. (E) RIP showing the association of HNRNPU with circ-UBE2K. Top, IP was performed using HMC3 cell lysates and either an IgG (control) antibody or an anti-HNRNPU antibody. IgG antibody served as a control. Bottom, the expression of circ-UBE2K in the pulled down material was measured by qPCR analysis. (F) IF-FISH assay showing that circ-UBE2K colocalized with the HNRNPU protein in HMC3 cells. Green, FITC-labeled probes specific for circ-UBE2K; green, HNRNPU protein; blue, DAPI (nuclei); the merged image shows the overlap of the fluorescent signals. (G-H) Representative Western blot images and relative expression of UBE2K. The data are presented as the means± SEM. from three independent experiments. ns, not significant, **P < 0.01. Unpaired t-test for E, One-way ANOVA followed by Tukey's post hoc test for H.

We further investigated the mechanism by which circ-UBE2K regulates the expression of UBE2K. First, a biotin-labeled probe covering the circ-UBE2K junction region was used for RNA pull-down analysis and mass spectrometry analysis to screen for circ-UBE2K-interacting proteins (**Figure 7A**). Mass spectrum analysis revealed that 10 proteins were pulled down by biotin probes targeting endogenous circ-UBE2K (**Figure 7B and Table S1**). GO analysis revealed that the main molecular functions of the pulled down proteins were “binding DNA and/or RNA”, “structural molecule activity”, “transporter activity”, and “catalytic activity” (**Figure 7C**). KEGG analysis further suggested that the enriched proteins play a key role in “neurodegenerative diseases” (**Figure S7**).

Among the enriched proteins, the protein HNRNPU had the greatest total protein expression and was found to play a potential role in RNA binding and selective RNA splicing (**Figure 7B**). Additionally, the GO terms found to be associated with this gene included RNA binding and kinase activity. Therefore, we used different assays to confirm the interaction between the HNRNPU protein and circ-UBE2K. First, we used biotin-labeled probes, streptavidin-coated beads and HMC3 cells lysates for pull-down analysis and then performed Western blot analysis of HNRNPU protein expression. As shown in **Figure 7D**, HNRNPU was pulled down by a circ-UBE2K-specific biotin probe but not a control biotin probe. Further RNA binding protein immunoprecipitation (RIP) assays revealed that the level of circ-UBE2K in the precipitate pulled down with the anti-HNRNPU antibody was significantly greater than that in the precipitate pulled down with control IgG (**Figure 7E**). In addition, RNA fluorescence *in situ* hybridization (RNA-FISH) and immunofluorescence assays confirmed the colocalization of endogenous circ-UBE2K and HNRNPU in HMC3 cells. This finding was further confirmed by the increased colocalization of circ-UBE2K and HNRNPU in the nucleus of HMC3 after LPS stimulation (**Figure 7F**). These findings suggested that circ-UBE2K interacted directly with the HNRNPU protein in HMC3 cells. Finally, we designed an HNRNPU siRNA to investigate whether HNRNPU knockdown can eliminate the positive regulatory effect of circ-UBE2K on UBE2K expression. As shown in **Figure 7G-H**, overexpression of circ-UBE2K increased the protein expression of the host gene UBE2K, while knockdown of HNRNPU reversed this change. In addition, we also tested whether knocking down HNRNPU can eliminate the increase in inflammatory factors caused by circ-UBE2K. The results showed that overexpression of circ-UBE2K, IL-6, and IL-1 β significantly increased in the presence or absence of LPS stimulation, and knocking down HNRNPU could eliminate these changes (Figure S8).

## Discussion

In the present study, our data revealed that a novel circRNA, circ-UBE2K (hsa_circ_0009154), is upregulated in the peripheral blood of patients with MDD and the brains of CUMS model mice. We present evidence that circ-UBE2K significantly exacerbates depressive-like behavior in a CUMS mouse model, leading to abnormal activation of microglia and neuroinflammation-related damage. Downregulation of circ-UBE2K may thus represent a potential therapeutic target for depression. Mechanistically, we found that circ-UBE2K interacts with the HNRNPU protein to regulate UBE2K protein expression, mediate abnormal microglial activation, and promote the progression of depression. Our findings link the epigenetic regulation of depression with abnormal activation of microglia and circ-UBE2K, providing evidence that circ-UBE2K may be a new therapeutic target for MDD.

The specific pathogenesis of depression is not yet clear, and its etiology is multifactorial 27. Previous studies have shown that circular RNAs play a role in the occurrence and development of depression 14, 25, 28, 29. and circular RNAs have high stability and are easy to detect in blood and other body fluids, making them ideal targets for disease diagnosis and treatment 30. This prompted us to perform transcriptome sequencing of blood samples collected from patients with MDD and healthy subjects. After database screening, qPCR validation, and clinical correlation analysis, it was found that circ-UBE2K was highly expressed in blood samples from patients with depression. Moreover, the level of circ-UBE2K in the peripheral blood of MDD patients was positively correlated with the HAMD-17 and HAMD-24 scores, which are important indicators for evaluating the occurrence of MDD. Consistent with previous human studies, circ-UBE2K was highly expressed in the brain tissue and peripheral blood of CUMS-induced depression model mice. Our findings suggest that an imbalance in circ-UBE2K levels in peripheral blood is associated with the pathophysiology of MDD.

Inflammation is closely related to the onset of depression, and inflammation in the central nervous system is one of the important pathological and physiological mechanisms that leads to depression 18. Microglia, as tissue-specific macrophages of the central nervous system (CNS), play a crucial regulatory role in immune and inflammatory processes in the nervous system 31. Recent studies have shown that microglia can regulate neuronal activity, promote learning, and shape social behavior 32-34. Activation of microglia, increase of cytokines and chemokines, recruitment of peripheral immune cells, and local tissue damage are observed in pathological environments such as injury and disease 35, 36. Evidence from clinical, animal, and *in vitro* studies suggests that the activation of microglia plays an important role in the pathophysiology of depression 20, 37. Consistent with these findings, in our study, GO enrichment analysis of the DEGs identified by high-throughput sequencing of peripheral blood samples from patients with depression revealed that the expression of genes associated with the inflammatory response was upregulated. The FISH-IF results showed that circ-UBE2K had the greatest expression in microglia in the mouse brain. Furthermore, our study revealed that CUMS treatment led to abnormal microglial activation and neuroinflammation, resulting in neuronal damage. Increasing the expression of circ-UBE2K aggravated neuroinflammation and neuronal damage. The array results showed that the expression levels of proinflammatory factors in the brain tissue of mice overexpressing circ-UBE2K increased. After injection of a microglia-specific adeno-associated virus and treatment with LPS, immunofluorescence, Western blotting and qPCR showed that downregulating circ-UBE2K could alleviate microglial activation and depression-like behavior in mice and reduce the expression of related inflammatory proteins. Based on these findings, abnormal activation of microglia during the development of depression may lead to subsequent neuroinflammation-related glial dysfunction. Classically identifying microglial activation through significant increase in cytokine production, adoption of amoeba morphology, and changes in related protein markers, these functions collectively represent various aspects of microglial activation 38, 39. Therefore, this study focused on microglia, and the *in vitro* results were validated through *in vivo* experiments to further explore the possible pathways and downstream targets by which circ-UBE2K regulates microglia. Consistent with other related studies, LPS treatment significantly increased the levels of proinflammatory cytokines (including IL-6, IL-1β and TNF-α) in microglia 40, 41. Circ-UBE2K siRNA significantly decreased the levels of proinflammatory cytokines produced by LPS-induced microglial activation.

The same circRNA has different functions in different diseases. Circ-UBE2K is highly expressed in bladder cancer and promotes tumor progression by functioning as a ceRNA to regulate ARHGAP5 expression by sponging miR-516b-5p 42. In this study, we determined that the expression of the circ-UBE2K parent gene was positively correlated with that of circ-UBE2K in human peripheral blood samples and mouse brain tissue and that overexpression of circ-UBE2K increased the expression of the UBE2K protein. The circUBE2K host gene ubiquitin binding enzyme E2K (UBE2K) is an E2 ubiquitin binding enzyme in the ubiquitin proteasome (UPS) pathway and is highly expressed in the mammalian brain. Studies have reported that the expression level of UBE2K is significantly altered in neurological diseases such as schizophrenia 43, Parkinson's disease 44 and chronic stress 45. Based on this evidence, we speculated that upregulated circ-UBE2K regulates the expression of host genes and is involved in the occurrence and development of MDD.

Many studies have shown that circRNAs can interact with their parental genes via epigenetic control, splicing, transcription, competitive binding, or translation 46. For example, circ-CUX1 promotes the interaction between CUX1 and MYC-associated zinc finger protein (MAZ) by binding to EWS RNA-binding protein 1 (EWSR1), altering the transactivation of MAZ, the transcription of CUX1 and other genes related to tumor progression, and promoting tumor occurrence and invasion 47. Reut Ashwal-Fluss et al. reported that there is a strong and direct interaction between the splicing protein MBL and circMBL. When the MBL protein is expressed at excess levels, it reduces its own mRNA production by promoting the production of circMBL. By binding to the MBL protein, circMBL removes excess MBL protein 48. A study by Liang et al. revealed that circS100A11 promotes S100A11 translation by competitively binding to CAPRIN1 to decrease the suppressive effect of CAPRIN1 on S100A11 translation 49. Given these complexities, we confirmed that circUBE2K binds to HNRNPU using RNA pull-down and mass spectrometry, RNA-binding protein immunoprecipitation assays, FISH and immunofluorescence staining. Previous reports have shown that HNRNPU, which binds to DNA and RNA elements, plays a role in transcription, alternative mRNA splicing and mRNA stability 50-52. HNRNPU increases the expression of TNF-α by regulating mRNA stability through binding to the 3'-untranslated region (UTR) 53. Further experiments confirmed that knockdown of HNRNPU significantly inhibited the positive regulation of UBE2K expression by circ-UBE2K.

In summary, the findings of the present study revealed that the expression of circUBE2K, which combines with HNRNPU to form the circUBE2K/HNRNPU complex, is increased in microglia after external stress, thus regulating the expression of the parental gene UBE2K and mediating the abnormal activation of microglia to induce neuroinflammation, promoting the development of MDD. This study provides new evidence that circ-UBE2K alleviates depressive-like behavior through the regulation of microglial function and suggests that circ-UBE2K may serve as a potential therapeutic target for the treatment of severe depression.

## Materials and Methods

### Ethics

The ethics committee of the Affiliated Hospital of Guangdong Medical University (No. YJYS2021070) approved this research protocol. All participants or their legal guardians provided written informed consent and were informed of the trial's benefits and risks. All animal studies are reported in accordance with the Animal Research: Reporting of *In Vivo* Experiments Guidelines and were reviewed and approved by the Institutional Animal Care and Use Committee at Guangdong Medical University (ID: GDY2102082).

### Animals

Adult male/female C57BL/6J mice (18.0-22.0 g, 4-5 weeks old) were purchased from GemPharmatech (Nanjing, China) and randomly divided into experimental groups. All mice were housed on a 12-hour light/dark cycle (lights on at 7 am) at a comfortable temperature (23-25°C), and given free access to water and food before the experimental procedure began. CUMS-exposed mice were housed in single cages, and control mice were housed in group cages.

### Plasmids, oligos and viruses

SiRNA oligonucleotides were designed and synthesized by HanBio (Shanghai, China). The sequences are listed in Supplementary **Table S2**. The circ-UBE2K-overexpressing plasmid was constructed by GeneChem (Shanghai, China). The overexpression plasmid Flag-HNRNPU was constructed by Yubo Biology (Shanghai, China). Lv-EF1-CMV-circ_UBE2K-ZsGreen was constructed and packaged by HanBio (Shanghai, China). AAV6-Iba1p-RNAi-circ-UBE2K-EGFP-SV40 was constructed and packaged by GeneChem (Shanghai, China).

### qPCR

RNA was extracted using TRIzol reagent (Invitrogen, USA) according to the manufacturer's protocol. Briefly, an Evo M-MLV RT Kit with gDNA Clean for qPCR II (AG Biotechnology, China) was used to perform reverse transcription. Next, the cDNA obtained in the previous step was used as the template for qPCR. qPCR was then performed on a LightCycler 96 (Roche Applied Science, Penzberg, Germany) using SYBR Green qPCR Master Mix (AG). The primers for circRNAs and mRNAs were synthesized by Sangon Biotech. The sequences of the primers used are listed in Supplementary **Table S3**.

### CUMS protocol

The CUMS protocol was modified from a previously validated protocol 54. The mice were exposed to unpredictable, randomly scheduled mild environmental stressors two times a day for 4 weeks. The stressors included (1) food or water deprivation for 24 hours, (2) overnight illumination, (3) lack of sawdust in the cage for 24 hours, (4) moistened sawdust in the cage for 24 hours, (5) red light flashing for 8 hours, (6) tail clipping (1 cm from the tip of the tail), (7) physical restraint for 6 hours and (8) 45° cage tilt along the vertical axis for 3 hours.

### Depressive-like behavior tests

Before the behavior tests, the mice were transferred to the test room for at least half a day. All tests were conducted under relatively quiet and low-intensity light conditions to minimize anxiety in the test mice. The researchers who performed the behavioral tests were blinded to the treatment groups.

### OFT

Mice were preconditioned to the testing environment. For testing, the mice were placed in a plastic chamber with a width of 40 cm, length of 40 cm and height of 40 cm for 5 min, and their behavior was recorded using a video-tracking system. The total distance traveled was recorded by software (YUYAN INSTRUMENT, Shang Hai). After each trial, the box was wiped with 75% ethanol to remove olfactory cues.

### SPT

Prior to beginning testing, the mice were provided with two identical drinking bottles for adaptation for 3 days. Then, the animals were deprived of water for 24 hours, and the mice were given a bottle of water and a bottle of 1% sucrose solution for 3 days; the positions of the two bottles were switched every day. Sucrose preference was calculated as the percentage of sucrose intake relative to total liquid intake, and the average value was calculated for each of the 3 days of the test. Sucrose preference was calculated as a percentage as follows: [(sucrose intake/total intake) × 100].

### FST

The FST was performed in a transparent glass cylinder (20 cm in height and 25 cm in diameter) filled with 15 cm of water at 23-25°C. The mice were placed in the cylinder for a total of 6 minutes. The animals were allowed to adapt to the cylinder for the first 2 minutes, and the test was performed over the last 4 minutes. The immobility time of the mice was recorded. The whole test was recorded by video, and software was used to calculate the immobility time offline.

### TST

The mouse tail was fixed to a tail suspension bracket with tape, which was positioned approximately 1 cm from the end of the tail. The mice were tested for 6 minutes. The behavior of the mice in the first two minutes, i.e., the adaptation phase, was not analyzed, and the actual test was conducted over the final 4 minutes. The immobility time of the mice was recorded. The whole test was recorded by video, and software was used to calculate the immobility time offline.

### Stereotaxic injection of virus

All mice were weighed before the experiment and anesthetized with 2% avodine. Lentivirus (1.5 mL, 10^9^ viral genomes/mL) (HanBio, Shanghai, China) was microinjected bilaterally into the hippocampus of C57BL/6J mice at the following coordinates relative to bregma: anteroposterior, - 2.3 mm; mediolateral, ± 1.8 mm; and dorsoventral, - 2.2 mm. The mice were returned to the heating pad after injection into the two hemispheres until their body temperature and skin color returned to normal.

### Brain tissue preparation

The mice were deeply anesthetized with chloral hydrate with avodine and perfused with saline until the fluid was clear. The brains were removed rapidly and divided into two hemispheres. For immunohistochemistry and FISH, the brain tissues were postfixed in 4% paraformaldehyde solution at 4°C overnight and then immersed in 10%, 20% or 30% sucrose solution at 4°C for 2‒3 days for dehydration. Then, the brains were immersed in optimal cutting temperature (OCT) compound and stored at -20°C. For qPCR and Western blotting, the dissected tissues were snap-frozen in liquid nitrogen and stored at -80°C until use for biochemical analyses.

### FISH

FITC-labeled probes specific for circ-UBE2K (Ginkgo Biotechnology, Guangzhou, China; **Table S3**) were used for hybridization. Frozen brains were cut into 10 μm coronal slices with a cryostat (HM525 NX, Thermo Scientific) at -20°C. Then, the slices were fixed with 4% paraformaldehyde at room temperature for 10-15 min. They were washed with 2× SSC 3 times at room temperature. The tissue slices were treated with 20 µg/ml protein kinase K for 5 min in a 37°C wet box, and subsequently washed 3 times with 2× SSC. Prehybridization was performed at 37°C for 1 h in hybridization buffer (the hybridization buffer was preheated to 37°C). A total of 50 µL of the probe was applied to the target area on the slide, which was covered with a coverslip (22x22 mm) to seal the edge of the slide. Denaturation was performed at 65°C for 5 minutes, after which the sections were allowed to hybridize at 37°C overnight (12-18 h). The next day, the samples were washed twice with 2× SSC at 42°C away from light for 5 min/wash (the SSC was preheated to 42°C). Filter paper was used to absorb the excess liquid. Nuclei were counterstained with DAPI. Images were acquired on an Olympus FV3000 confocal microscope. The sequence of the probe was as follows: circ-UBE2K: 3′-FITC- TCTGTAAAATTCTCATCTACAAGATCTACTTTAATTTGATTTTTGCTCGT^CATTGATCTTTCAGGATATCCAAACAAATAGCCCCTGTGACGGAACT.

### Immunostaining

Frozen coronal sections were fixed with 4% paraformaldehyde for 5 min and incubated with Enhanced Immunostaining Permeabilization Solution (P0097, Beyotime Biotechnology, China) for 5 min. After being blocked with 10% goat serum for 30 min, the sections were incubated with primary antibody at 4°C overnight. On the second day, the sections were washed with PBS and stained with a fluorescent secondary antibody at room temperature for 1 hour. Nuclei were counterstained for 3 minutes with DAPI. Images were acquired on an Olympus FV3000 confocal microscope.

### Cell sorting

Cell sorting was performed using the Adult Brain Dissociation Kit (130-107-677, Miltenyi Biotec) according to the manufacturer's instructions. Briefly, the mice were killed by cervical dislocation, and the whole brains of the mice were removed and washed in cold D-PBS. The whole brains were cut into small pieces using a scalpel and then transferred to a gentleMACS C tube (# 130-093-237, Miltenyi Biotec) containing enzyme mixture 1. Then, enzyme mix 2 was added to the C tube, which was subsequently attached upside down in the sleeve of the gentleMACS Octo Dissociator with Heaters (# 130-096-427, Miltenyi Biotec) for dissociation for 1 hour. After termination of the reaction, the dispersed cells were passed through 70-μm nylon mesh (# 130-093-235, Miltenyi Biotec) and collected by centrifugation. Finally, the cells were resuspended to remove the cell fragments and red blood cells. Neurons (Neuron Isolation Kit, mouse, # 130-098-752, Miltenyi Biotec), astrocytes (Anti-ACSA-2 MicroBead Kit, mouse, # 130-097-678, Miltenyi Biotec) and microglia (CD11b (Microglia) MicroBeads, human and mouse, (# 130-093-634, Miltenyi Biotec) were isolated by the MACS technique.

### Western blotting

Cells or tissues were lysed with lysis buffer (P0013, Beyotime) containing protease inhibitor cocktail (4693116001, Sigma) and phenylmethylsulfonyl fluoride (PMSF) on ice, and the protein concentration was measured with a BCA assay (23335, Pierce). The proteins were separated by SDS‒PAGE and then transferred to a polyvinylidene fluoride (PVDF) membrane (Millipore, MA, USA). The PVDF membranes were blocked with 5% nonfat milk powder in TBST and incubated with specific primary antibodies overnight at 4 °C. Then, the membrane was incubated with a goat anti-rabbit or mouse IgG secondary antibody conjugated to HRP. The protein bands were detected using an enhanced chemiluminescence kit (Millipore, MA, USA) and visualized with an enhanced chemiluminescence (ECL) bioimaging system (Azure, USA). The density of each protein band was quantified using ImageJ software and normalized to the density of the α-tubulin band. Details about the antibodies used are listed in Supplementary **Table S4**.

### Golgi staining

Golgi staining was performed using the FD Rapid GolgiStain Kit (PK401, FD Neuro Technologies) according to the manufacturer's instructions and other studies 55. In brief, brain tissues were quickly stripped and transferred to Golgi Cox solution (A solution+B solution). After being stored at room temperature in the dark for 2 weeks, the brain tissues were transferred to tissue protectant solution (C solution) for 3 days. Then, the brains were cut into 150 mm coronal sections using a vibrating microtome (VT 1000S, Leica). The slices were immersed in a mixture of solution D, solution E and ddH2O (1:1:2) for 10 minutes, dehydrated in ethanol fractionated solution, cleaned in xylene solution, and sealed with neutral resin. Images were acquired on a laser microdissector (Carl Zeiss, Thornwood, NY) and analyzed using ImageJ software.

### Cytoplasmic and nuclear RNA purification

Cytoplasmic and nuclear RNA was isolated using the reagents supplied in the Cytoplasmic & Nuclear RNA Purification Kit (21000, Norgen Biotek) according to the manufacturer's instructions. Briefly, HMC3 cells were lysed in ice-cold lysis buffer J and centrifuged for 10 minutes at maximum speed in a benchtop centrifuge. The supernatant, which contained cytoplasmic RNA, was transferred to another RNase-free tube. After the pellet was washed with Cell Fraction Buffer, the nuclei were collected. Cytoplasmic and nuclear RNA was separated and purified for qPCR according to the manufacturer's instructions.

### RNA pull-down and mass spectrometry

According to the manufacturer's instructions, an RNA pull-down assay was conducted with an RNA pull-down positive kit (GzScbio, Guangzhou, China). First, a 5'-biotinylated circ-UBE2K probe was combined with streptavidin-labeled magnetic beads to form an RNA/magnetic bead complex, which was subsequently incubated with HMC3 cell lysates. The proteins were eluted with SDT buffer. Protein expression was analyzed by Western blotting or mass spectrometry (Shanghai Applied Protein Technology Co., Ltd.). The 5',3'-biotinylated circ-UBE2K probe was purchased from Ginkgo Biotechnology (Guangzhou, China). The sequence of the probe was as follows: circ-UBE2K: 3′-biotin- TCTGTAAAATTCTCATCTACAAGATCTACTTTAATTTGATTTTTGCTCGT^CATTGATCTTTCAGGATATCCAAACAAATAGCCCCTGTGACGGAACT- biotin -5'.

### RIP

RIP was performed by using the Magna RIP RNA-Binding Protein Immuno-Precipitation Kit (17-701, Merck Millipore, USA) according to the manufacturer's instructions. Briefly, HMC3 cells were harvested when the confluence reached 90% after 48 hours after transfection and were subsequently lysed in RIP lysis buffer. After centrifugation, the supernatant was incubated with beads bound to control mouse IgG or an anti-HNRNPU antibody at 4 °C overnight. The bound proteins were further analyzed by Western blotting. The immunoprecipitated RNA was used for qPCR analysis.

### Cell culture, transfection, and drug treatment

HMC3 cells were cultured under the recommended conditions. The cells were seeded in 6-well or 12-well plates and cultured to 60%-70% confluence before transfection. Plasmids were transiently transfected using Lipofectamine LTX (15338100, Thermo Fisher Scientific), and siRNAs were transiently transfected using Lipofectamine RNAiMAX (catalog number 13778150, Thermo Fisher Scientific). Transfection was performed according to the manufacturer's protocols.

### Statistical analysis

GraphPad Prism 8 (GraphPad Software, USA) was used for statistical analyses and graphing. ImageJ software was used to quantitatively analyze protein expression. FUJI software was used to quantitatively analyze the immunofluorescence images. The data are presented as the means ± standard errors of the means (SEMs). Statistical analyses were performed by Student's t test for comparisons between two groups. One/two-way ANOVA followed by the Holm‒Sidak test was used for multigroup (three or more) comparisons.

## Supplementary Material

Supplementary figures and tables 2-4.

Supplementary table 1.

## Abbreviations

circ-UBE2K: circular RNA UBE2K
MDD: major depressive disorder
HNRNPU: heterogeneous nuclear ribonucleoprotein U
UBE2K: ubiquitin conjugating enzyme E2 K
ROC: receiver operating characteristic
AUC: area under the curve
CUMS: chronic unpredictable mild stress
FST: forced swimming test
TST: tail suspension test
SPT: sugar preference test
DEP: differentially expressed proteins
PSD95: postsynaptic protein
FISH: fluorescence *in situ* hybridization
RIP: RNA binding protein immunoprecipitation
CNS: central nervous system
UPS: ubiquitin proteasome
