# Supplementary material for: Microglial circ-UBE2K exacerbates depression by regulating parental gene UBE2K via targeting HNRNPU
Source: Theranostics. 2024 Jul 1;14(10):4058–75. doi: 10.7150/thno.96890 (PMC11234284; doi:10.7150/thno.96890)
Supplement: Supplementary file 1 — Supplementary figures and tables 2-4. [file thnov14p4058s1.pdf]

Supplementary information

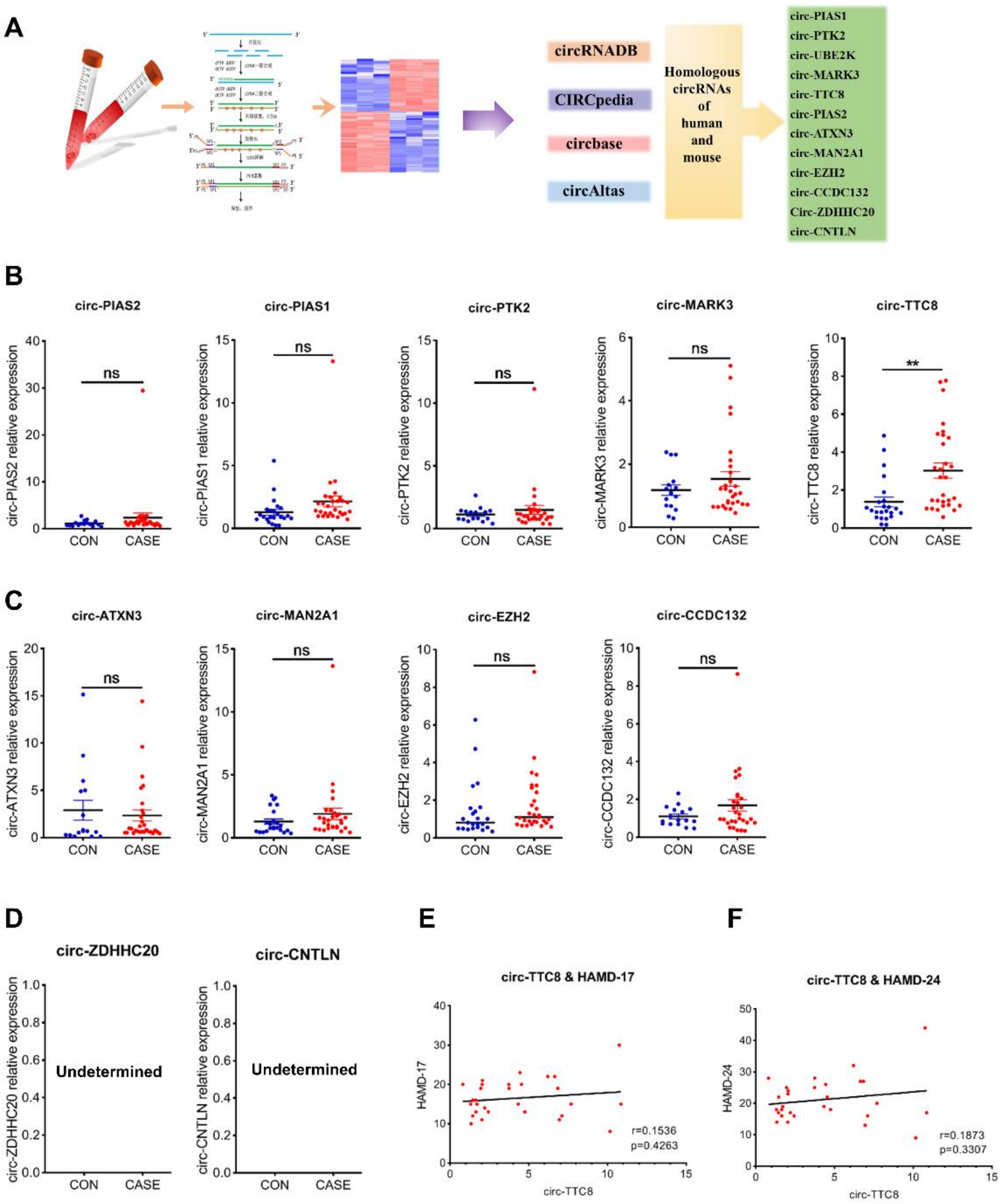

Figure S1. Validation of the high-throughput sequencing data by qPCR. (A) Schematic diagram of the transcriptome sequencing protocol and heatmap of differentially expressed circular RNAs in blood samples from patients with depression.(B-D) The differential expression of circRNAs with homology between

humans and mice identified by high-throughput sequencing of blood samples from depressed patients (n=29) and healthy control subjects (n=16) was verified by real-time PCR. (E) Correlations between circ-TTC8 expression and HAMD-17 scores were determined using Pearson's correlation coefficient. (F) Correlations between circ-TTC8 expression and HAMD-24 scores were determined using Pearson's correlation coefficient. The data are presented as the mean ± SEM. P values were determined by a two-tailed unpaired Student's t test. **$P < 0.01$.

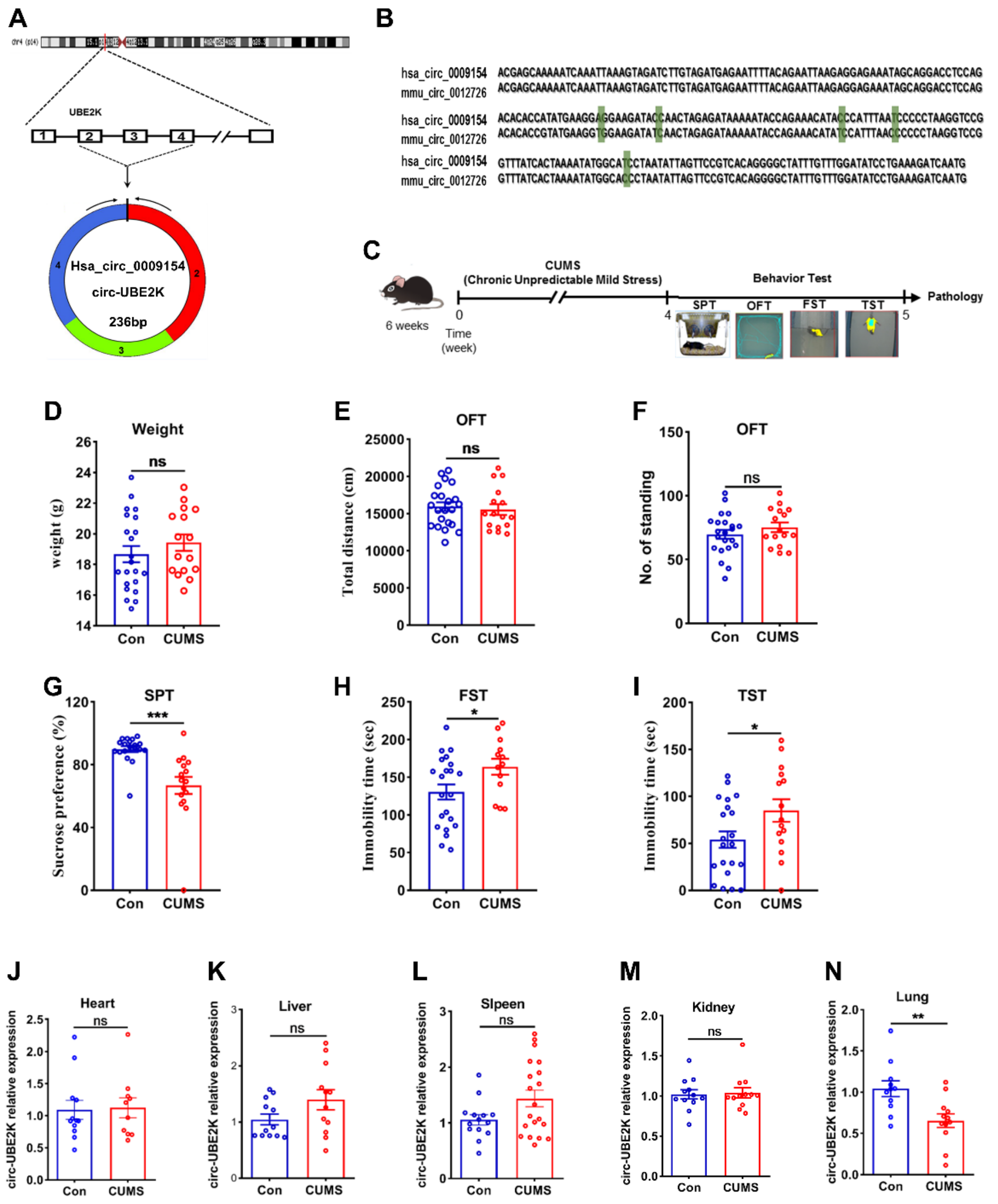

Figure S2. Construction of a depression mouse model and behavioral analysis. (A) Schematic diagram of circ-UBE2K. (B) Analysis of circ-UBE2K sequence alignment between humans and mice. (C) Schematic diagram of the mouse model and behavioral tests. (D) Body weight changes in mice 4 weeks after CUMS treatment. (E-F) The total distance traveled (E) and number of standing of mice from different groups in the OFT. (G-I) Performance of CUMS model mice and control mice in the behavioral tests (SPT, FST and TST). (J-N) The expression levels of circ-UBE2K in the heart, liver, spleen,

lung and kidney of CUMS-induced depression model mice and control mice. n=13-22/group. The data are presented as the mean ± SEM. P values were determined by a two-tailed unpaired Student's t test. ns, not significant, *$P < 0.05$, **$P < 0.01$ and ***$P < 0.001$.

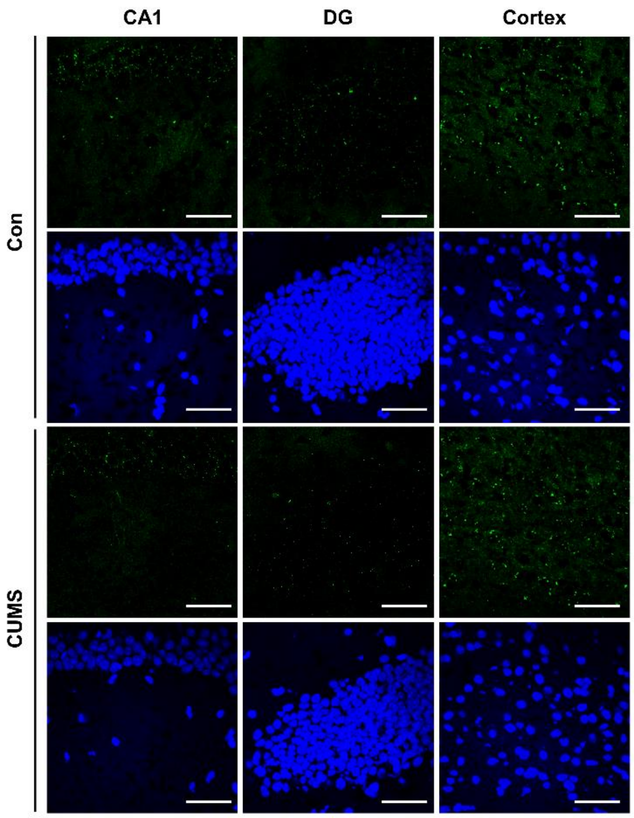

Figure S3. Circ-UBE2K was significantly upregulated in depression model animals. Images of circ-UBE2K in brain tissues from CUMS model mice and control mice. Green, FITC-labeled probes specific for circ-UBE2K; blue, DAPI (nuclei). Scale bar, 50 μm.

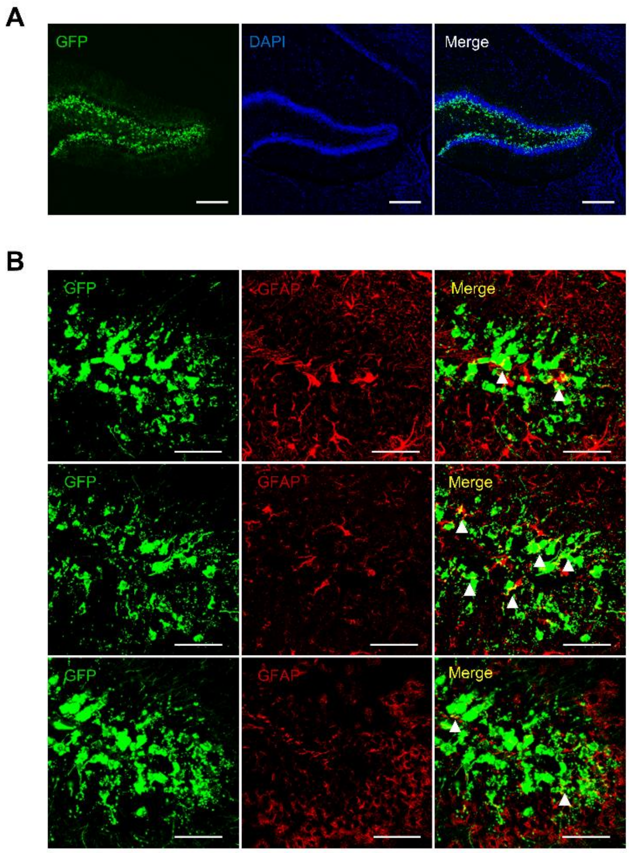

Figure S4. Microinjection of circ-UBE2K lentivirus into the hippocampus. (A) Evaluation of circ-UBE2K expression by analysis of GFP expression at 4 weeks after microinjection. Scale bar, 50 μm. (B) Distribution of the circ-UBE2K lentivirus in different cell types in the hippocampus 4 weeks after lentivirus microinjection. The white triangular arrow points to the co-localization of circ-UBE2K with astrocytes, microglia and neurons. Scale bar, 50 μm.

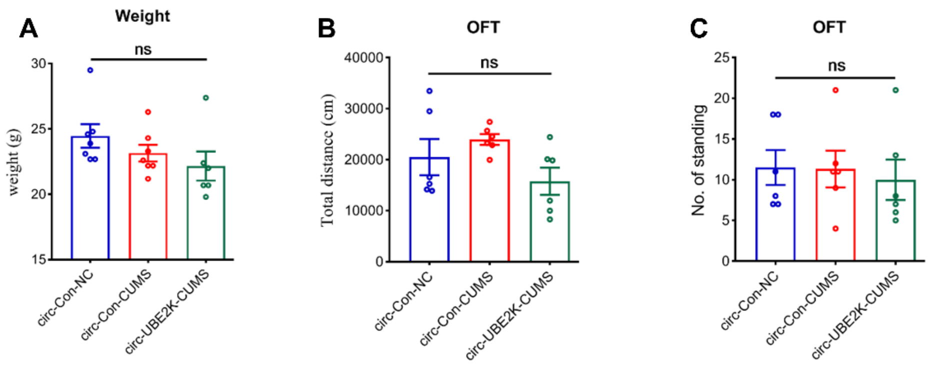

Figure S5. Overexpression of circ-UBE2K aggravated depression-like behavior. (A) Body weight changes in mice 4 weeks after CUMS treatment. (B) The total distance traveled of mice from different groups in the OFT. (C) The number of standing of mice from different groups in the OFT. One-way ANOVA followed by Tukey’s post hoc test. All the data are presented as the mean ± SEM. ns, not significant.

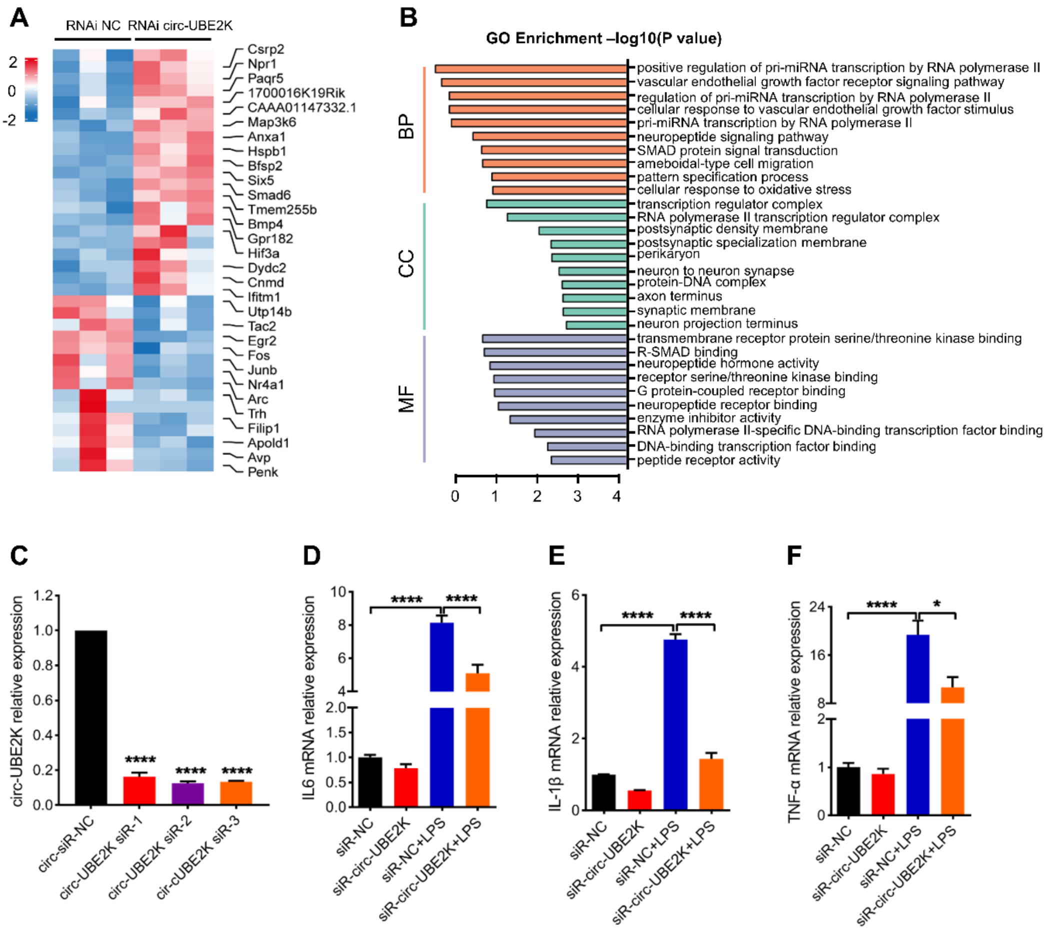

Figure S6. Knockdown of circ-UBE2K inhibits LPS-induced microglial secretion of inflammatory factors. (A) Heatmaps of the DEGs. (B) GO enrichment analysis of the enriched biological processes for important DEGs. (C) Relative expression of circ-UBE2K in microglia transfected with circ-UBE2K siRNA. (D-F) The mRNA expression levels of IL-6, IL-1β and TNF-α were measured by qPCR. The data are presented as the means ± S.D. from three independent experiments. One-way ANOVA followed by Tukey's post hoc test. **P* < 0.05 and *****P* < 0.0001.

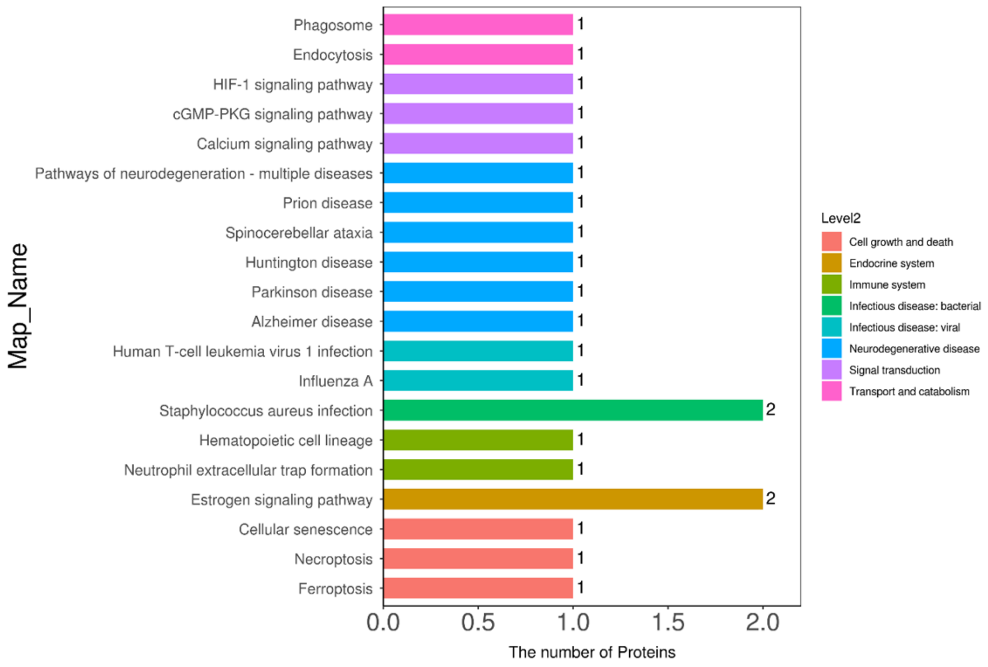

Figure S7. KEGG pathway analysis of the pulled-down proteins.

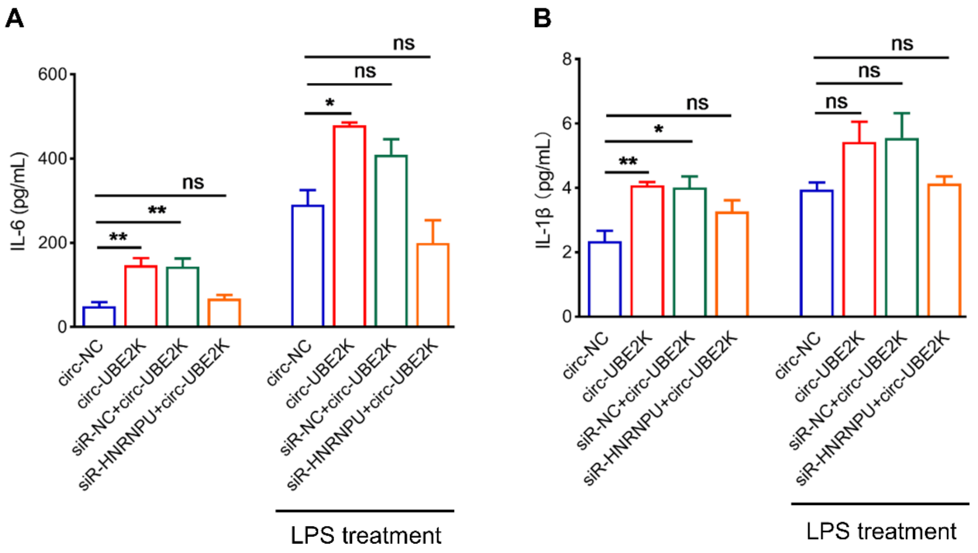

Figure S8. Circ-UBE2K directly interacts with the HNRNPU protein in microglia. (A) The levels of the proinflammatory cytokines IL-6 in the different groups. (B) The levels of the proinflammatory cytokines IL-1β in the different groups. ns, not significant, **P* < 0.05 and ***P* < 0.01.

Supplemental Tables

Table S2. Nucleotide sequences used in this study.

siRNA sequences	5'-3'
siRNA NC sense	UUCUCCGAACGUGUCACGU TT
siRNA NC antisense	ACGUGACACGUUCGGAGAA TT
circ-UBE2K-si-1 sense	AAGAUCAAUGACGAGCAAAAA TT
circ-UBE2K-si-1 antisense	UUUUUGCUCGUCAUUGAUCUU TT
circ-UBE2K-si-2 sense	AUCAAUGACGAGCAAAAAUCATT
circ-UBE2K-si-2 antisense	UGAUUUUUGCUCGUCAUUGAU TT
circ-UBE2K-si-3 sense	GAAAGAUCAAUGACGAGCAAATT
circ-UBE2K-si-3 antisense	UUUGCUCGUCAUUGAUCUUUC TT
Human HNRNPU-si-1 sense	CAGUGCUUCUUCCCUUACAAU TT
Human HNRNPU-si-1 antisense	AUUGUAAGGGAAGAAGCACUGTT
Human HNRNPU-si-2 sense	GCAACUGUGAGACUGAAGAUU TT
Human HNRNPU-si-2 antisense	AAUCUUCAGUCUCACAGUUGC TT
Human HNRNPU-si-3 sense	AGAUCAUGGCCGUGGAUAUUU TT
Human HNRNPU-si-3 antisense	AAAUAUCCACGGCCAUGAUCU TT

Table S3. Information on the PCR primers.

List of oligonucleotide sequences	5'-3'
circ-PIAS1-F	AGCTTTAACATCAGACAACAGTC
circ-PIAS1-R	TGTTGTGGTGTCAAGGCAAA
circ- PTK2-F	ATCATACTGGGAGATGCGGG
circ-PTK2-R	TGCTAGGTATCTGTCATATTTCT
circ-MARK3-F	ACAATCGGCAAGGGGAATTT
circ-MARK3-R	CATGTGACGTGTGCTTTTGT
circ-UBE2K-F	TTCCGTCACAGGGGCTATTT
circ-UBE2K-R	TTTTGCTCGTCATTGATCTTTCA
circ-TTC8-F	AGTAACCCTGCTCTGTGGAA
circ-TTC8-R	AGCCAGATCCAAAGCCTCAT
circ-PIAS2-F	GCCCACGAGTTTAGAATATGGT
circ-PIAS2-R	CATCAGGAGGTCATGCTTGC
circ-MAN2A1-F	TCACAAGATTCTCTGCCACAA
circ-MAN2A1-R	AACTGTATCTTGGCTCCGCA
circ-EZH2-F	ACTTCTAATAATCATGGGCCAGAC
circ-EZH2-R	AGCTCGTCTGAACCTCTTGA
circ-CCDC132-F	TGTGGATGAGCAGACAGGAG
circ-CCDC132-R	CATCGAGCCGTGTTCAACAA
circ-ATXN3-F	CTCTCTTGACGGGTCCAGAA
circ-ATXN3-R	GCCTTCTTGCTTCCTGTTGT
Human GAPDH-F	ACAACTTTGGTATCGTGGAAGG
Human GAPDH-R	GCCATCACGCCACAGTTTC
Mouse GAPDH-F	AGGTCGGTGTGAACGGATTTG
Mouse GAPDH-R	TGTAGACCATGTAGTTGAGGTCA
Human UBE2K-F	GTTCCGTCACAGGGGCTATTT
Human UBE2K-R	AATACCGTGCGGAGAGTCATT
Mouse IL-6-F	GGCGGATCGGATGTTGTGAT
Mouse IL-6-R	GGACCCCAGACAATCGGTTG
Mouse IL-1β-F	TGCCACCTTTTGACAGTGATG
Mouse IL-1β-R	TGATGTGCTGCTGCGAGATT
Mouse TNFα-F	TGTGCTCAGAGCTTTCAACAA
Mouse TNFα-R	CTTGATGGTGGTGCATGAGA
Mouse iNOS-F	AGGGCTTGGCTGAGTGAG
Mouse iNOS-R	GAGCGAGTTGTGGATTGTC
Mouse CD68-F	CTGGCTGTGCTTTCTGTG
Mouse CD68-R	TCTTGCTAGGACCGCTTAT
Human U3-F	TTCTCTGAGCGTGTAGAGCACCGA
Human U3-R	GATCATCAATGGCTGACGGCAGTT
Human β-actin-F	GGGAAATCGTGCGTGACATTAAG
Human β-actin-R	TGTGTTGGCGTACAGGTCTTTG
Human IL-1β-F	AGCTACGAATCTCCGACCAC

Human IL-1β-R	CGTTATCCCATGTGTCGAAGAA
Human TNFα-F	GAGGCCAAGCCCTGGTATG
Human TNFα-R	CGGGCCGATTGATCTCAGC
Human IL-6-F	CCTGAACCTTCCAAAGATGGC
Human IL-6-R	TTCACCAGGCAAGTCTCCTCA

Table S4. Details of the antibodies.

Antibodies	Brand	Catalog Number	Application
Rabbit anti-Iba1	Wako	019-19714	IF
Rabbit anti-a-Tublin	Abcam	ab4074	WB
Rabbit anti-CD68	Abcam	ab125212	IF, WB
Rabbit anti-UBE2K	Abcam	ab52930	WB
Rabbit anti-iNOS	Proteintech	18985-1-AP	IF, WB
Rabbit anti-Iba1	Wako	016-20001	WB
Rabbit anti-hnRNP U	Abcam	ab180952	IF,WB
Rabbit anti-GFAP	Abcam	ab7260	IF
Rabbit anti-MAP2	Cell Signaling Technology	8707S	IF
Rabbit anti-PSD95	Cell Signaling Technology	3450S	WB
Rabbit anti-Synatophysin	Proteintech	17785-1-AP	WB
Rabbit anti-IL-6	Abcam	ab259341	WB
Mouse anti-TNF Alpha	Proteintech	60291-1-Ig	WB
Rabbit anti-IL-1 beta	Abcam	ab283818	WB
Goat Anti-Rabbit 488	Abcam	ab150077	IF
Goat Anti-Rabbit 594	Abcam	ab 150080	IF
Goat Anti-Rabbit 647	Abcam	ab 150079	IF
